# Machine Learning Prediction Models to Evaluate the Strength of Recycled Aggregate Concrete

**DOI:** 10.3390/ma15082823

**Published:** 2022-04-12

**Authors:** Xiongzhou Yuan, Yuze Tian, Waqas Ahmad, Ayaz Ahmad, Kseniia Iurevna Usanova, Abdeliazim Mustafa Mohamed, Rana Khallaf

**Affiliations:** 1 School of Traffic and Environment, Shenzhen Institute of Information Technology, Shenzhen 518172, China; couscous_yuan@sina.com; 2 School of Civil Engineering, University of Science and Technology Liaoning, Anshan 114051, China; 3 Department of Civil Engineering, COMSATS University Islamabad, Abbottabad 22060, Pakistan; ayazahmad@cuiatd.edu.pk; 4 MaREI Centre, Ryan Institute, School of Engineering, College of Science and Engineering, National University of Ireland, H91 TK33 Galway, Ireland; 5 Peter the Great St. Petersburg Polytechnic University, 195291 St. Petersburg, Russia; usanova_kyu@spbstu.ru; 6 Department of Civil Engineering, College of Engineering in Al-Kharj, Prince Sattam Bin Abdulaziz University, Al-Kharj 11942, Saudi Arabia; a.bilal@psau.edu.sa; 7 Building & Construction Technology Department, Bayan College of Science and Technology, Khartoum 11115, Sudan; 8 Structural Engineering and Construction Management, Faculty of Engineering and Technology, Future University in Egypt, New Cairo 11845, Egypt; rana.khallaf@fue.edu.eg

**Keywords:** recycled aggregate concrete, sustainable aggregate, compressive strength, flexural strength, gradient boosting, random forest

## Abstract

Compressive and flexural strength are the crucial properties of a material. The strength of recycled aggregate concrete (RAC) is comparatively lower than that of natural aggregate concrete. Several factors, including the recycled aggregate replacement ratio, parent concrete strength, water–cement ratio, water absorption, density of the recycled aggregate, etc., affect the RAC’s strength. Several studies have been performed to study the impact of these factors individually. However, it is challenging to examine their combined impact on the strength of RAC through experimental investigations. Experimental studies involve casting, curing, and testing samples, for which substantial effort, price, and time are needed. For rapid and cost-effective research, it is critical to apply new methods to the stated purpose. In this research, the compressive and flexural strengths of RAC were predicted using ensemble machine learning methods, including gradient boosting and random forest. Twelve input factors were used in the dataset, and their influence on the strength of RAC was analyzed. The models were validated and compared using correlation coefficients (R^2^), variance between predicted and experimental results, statistical tests, and k-fold analysis. The random forest approach outperformed gradient boosting in anticipating the strength of RAC, with an R^2^ of 0.91 and 0.86 for compressive and flexural strength, respectively. The models’ decreased error values, such as mean absolute error (MAE) and root-mean-square error (RMSE), confirmed the higher precision of the random forest models. The MAE values for the random forest models were 4.19 MPa and 0.56 MPa, whereas the MAE values for the gradient boosting models were 4.78 MPa and 0.64 MPa, for compressive and flexural strengths, respectively. Machine learning technologies will benefit the construction sector by facilitating the evaluation of material properties in a quick and cost-effective manner.

## 1. Introduction

Numerous tests are performed to measure concrete performance, but compressive strength is frequently considered the most significant [1]. Compressive strength tests offer good insight into the concrete’s diverse properties. The compressive strength of concrete is directly or indirectly connected to a number of mechanical and durability properties [2]. Flexural strength is also a key characteristic to consider when designing structural concrete, since it has an effect on the flexural cracking, shear strength, deflection properties, and brittleness ratio of the concrete [3]. The compressive and flexural strength of recycled aggregate concrete (RAC) are reliant on a number of variables, including the mechanical and physical properties of the recycled aggregate used, as well as the microstructure of the resulting matrix [4]. Typically, RAC has an inferior compressive and flexural strength compared to natural aggregate concrete, owing to insufficient bonding between the aggregate and the old mortar, fractures and cracks in the recycled aggregate formed during the recycling procedure, and the presence of low-permeability mortar connected to the recycled aggregate [5,6,7]. Furthermore, the characteristics of RAC are reliant on the amount of recycled aggregate substituted and the moisture content [8,9]. The strength of RAC varies according to the recycled aggregate replacement ratio, the water–cement ratio (w/c), the recycled aggregate’s moisture content, and the recycled aggregate’s physical and mechanical properties [9,10]. When w/c is held constant, experimental data suggest that recycled aggregate replacement content has a significant effect on the strength of RAC [11,12]. When natural aggregate is totally replaced with recycled aggregate, the compressive strength of RAC can be reduced by up to 30% [13,14]. Similarly, other researchers discovered a drop in compressive strength of between 12% and 25% with 100% recycled aggregate incorporation [15,16]. It was discovered that the age of the waste concrete from which the recycled aggregate was manufactured had a substantial impact on the strength of the RAC [17]. Moreover, the strength of the parent concrete from which recycled aggregates are produced affects the strength of the RAC [18]. Hence, there are several factors that influence the strength of RAC, and to study their combined impact through experimental investigations is challenging. In contrast, using computational methods might better examine the combined influence of these factors on the strength of RAC.

The practice of developing models for forecasting the strength of concrete is ongoing in order to reduce unnecessary test repetitions and material waste. There are several prominent models for modeling concrete properties, such as best fit curves (based on regression analysis). However, due to the nonlinear behavior of concrete [19,20], regression models generated using this technique may not accurately represent the underlying nature of the material. Additionally, regression methods may understate the effect of constituent materials in concrete [21]. Artificial intelligence techniques such as machine learning are some of the more contemporary modeling techniques that have been used in the area of civil engineering. These approaches use input parameters to model responses, and the output models are validated by experimentation. For construction applications, machine learning algorithms estimate concrete strength [22,23,24,25,26], bituminous mixture performance [27], and concrete durability [28,29,30].

This study concentrates on the use of machine learning methods to forecast the compressive and flexural strength of RAC. Two distinct ensemble machine learning techniques were used—gradient boosting and random forest—and the effectiveness of both methods was evaluated using correlation coefficients (R^2^) and statistical checks. Moreover, k-fold analysis and error distributions were used to determine the validity of each technique. The reason for selecting the ensemble machine learning method was that the literature reported their better performance compared to individual machine learning methods, such as support-vector machines and artificial neural networks [31,32,33]. This research is interesting in that it predicts both the compressive and flexural strength via two ensemble machine learning methods, while experimental studies require considerable human effort, the cost of experimentation, and time for material collection, casting, curing, and testing. Since a number of factors—including w/c, recycled aggregate replacement ratio, parent concrete strength, water absorption of the recycled aggregate, density of the recycled aggregate, etc.—influence the strength of RAC, their combined impact is hard to study through an experimental approach. Machine learning methods are capable of determining their combined impact at a reduced effort. Machine learning methods require a dataset, which may be collected from past studies, since many investigations have been undertaken to determine material strength, and such a dataset might be utilized for training the machine learning models and forecasting the material properties. The purpose of this work is to ascertain the most appropriate machine learning method for the compressive and flexural strength estimation of RAC based on the results forecast and the effects of various parameters on the strength of RAC.

## 2. Methods

### 2.1. Data Retrieval and Analysis

To obtain the appropriate result, supervised machine learning techniques need a varied range of input variables [34,35,36]. The compressive and flexural strength of RAC were projected using data obtained from the past studies (see Table S1 in Supplementary Materials). Experimental data were arbitrarily selected from previous studies so as to avoid biased images. Twelve variables were chosen as input factors, as listed below:

- Recycled concrete aggregate (RCA) replacement ratio;
- Parent concrete strength, bulk density of natural aggregate;
- Bulk density of RCA;
- Bulk density of natural aggregate;
- Water absorption of natural aggregate;
- Water absorption of RCA;
- Aggregate–cement ratio (a/c);
- Effective water–cement ratio (w_eff_/c);
- Nominal maximum natural aggregate size;
- Nominal maximum RCA size;
- Los Angeles abrasion index of natural aggregate;
- Los Angeles abrasion index of RCA.

**Figure 1:**
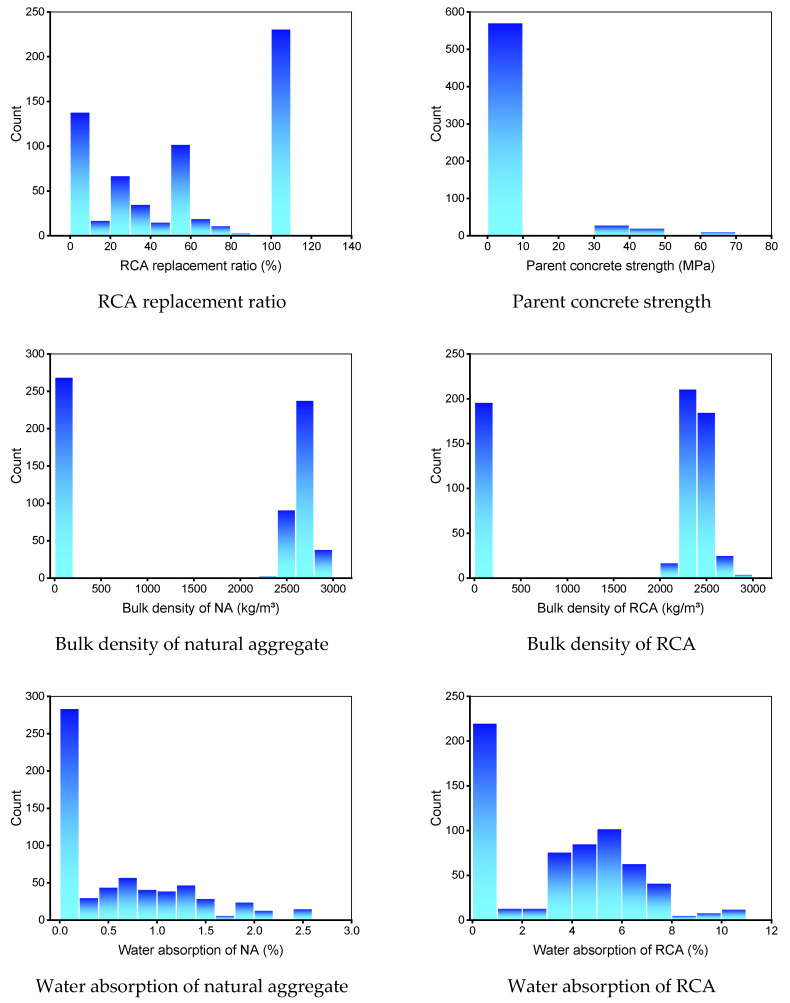

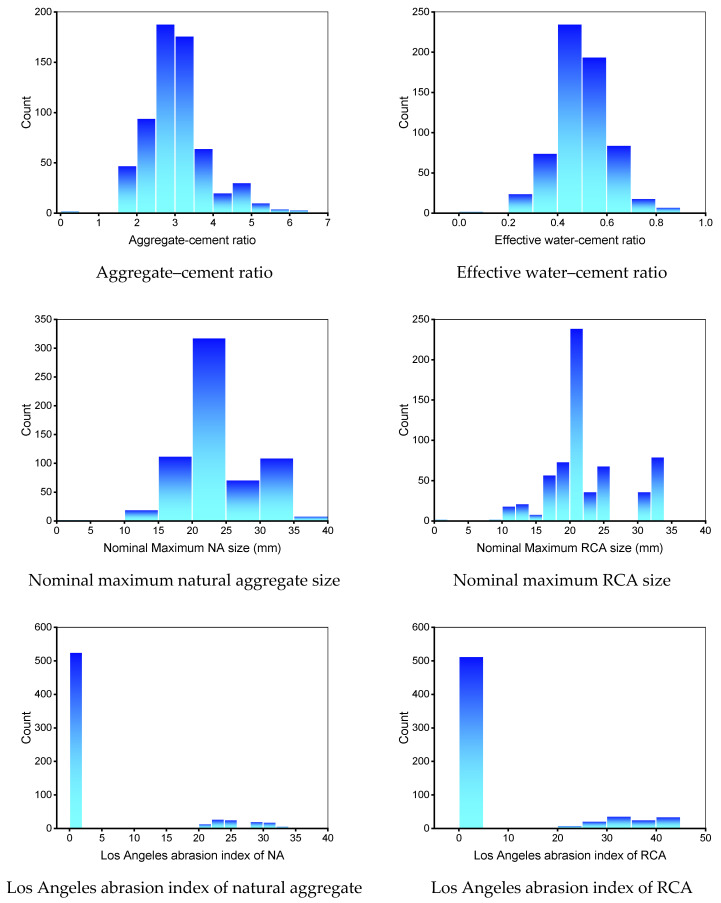
Relative frequency dispersal of input parameters for the compressive strength dataset. NA: natural aggregate, RCA: recycled concrete aggregate.

**Figure 2:**
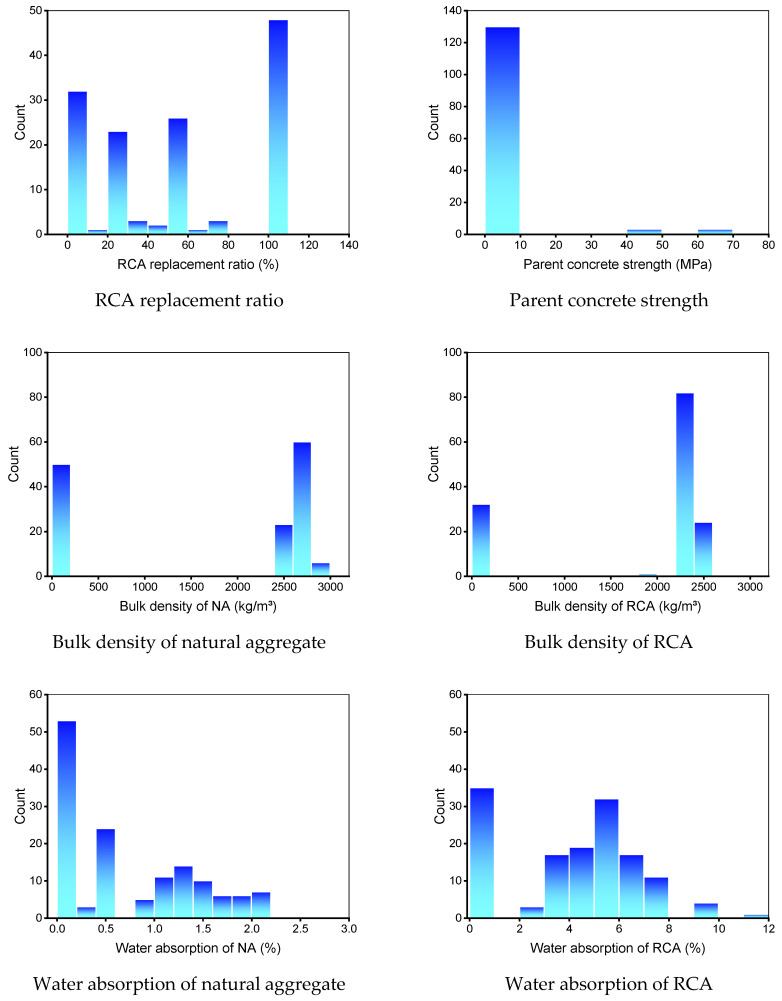

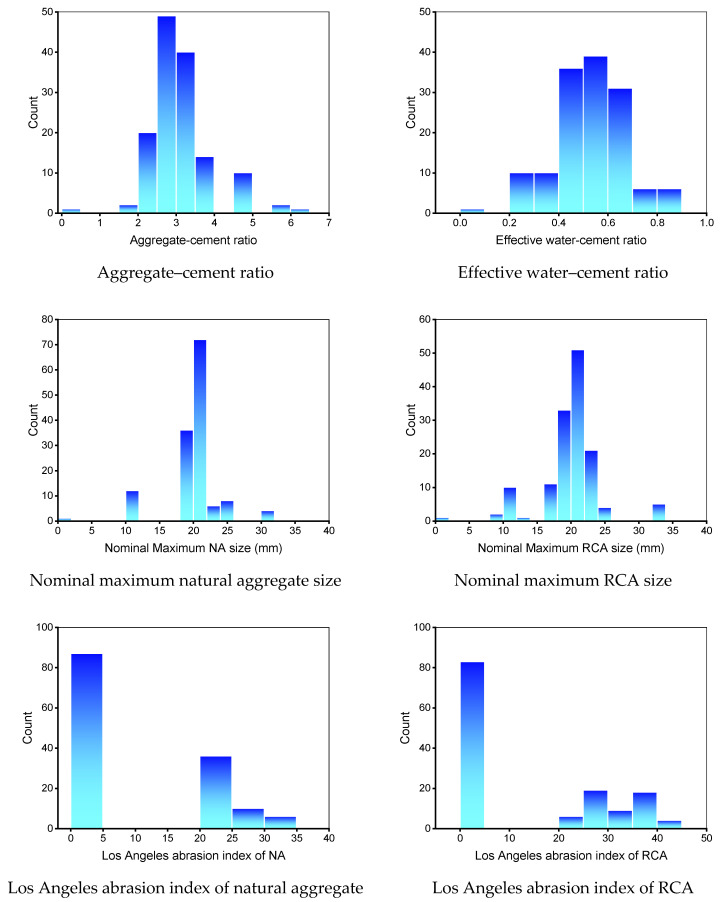
Relative frequency dispersal of inputs parameters for the flexural strength dataset. NA: natural aggregate, RCA: recycled concrete aggregate.

In addition, the compressive and flexural strength were chosen as the output variables. The quantity of input variables and the dataset have a substantial impact on a machine learning method’s result [37,38,39]. In the present study, 638 data points (mixes) were employed to run machine learning methods for compressive strength prediction, and 139 data points (mixes) were used for flexural strength prediction. Table 1 and Table 2 summarize the descriptive statistic evaluation of each input variable for compressive and flexural strength prediction, respectively. The mode, median, and mean exemplify basic propensity, while the standard deviation, minimum, and maximum denote variability. The entries in Table 1 and Table 2 with 0 (zero) do not represent actual physical measurements. Instead, these zero values indicate unavailable data from the original studies. The relative frequency dispersal of input factors employed to forecast the compressive and flexural strength is depicted in Figure 1 and Figure 2, respectively. This represents the overall number of readings linked to each input parameter.

### 2.2. Machine Learning Methods Employed

**Figure 3:**
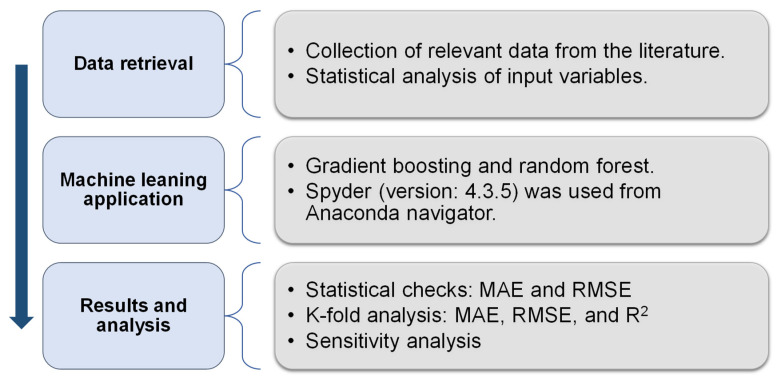
Flowchart of research methods.

Two ensemble machine learning methods (gradient boosting and random forest) were used to accomplish the objectives of this research, using Python code and the Anaconda Navigator program. Spyder 4.3.5 was used to execute the gradient boosting and random forest methods. Typically, these machine learning methods are used to anticipate desired outputs based on input factors. These methods are capable of forecasting the temperature effects, the strength properties, and the durability of materials [40,41]. Ensemble machine learning methods commonly exploit the weak learner by constructing 20 submodels that may be trained on data and modified to maximize the R^2^ value. The strategies to choose optimal hyperparameters include splitting the data for training and testing models (80% for training and 20% for testing), selecting the optimal submodel based on R^2^, and the k-fold analysis method. R^2^ represents the performance/validity of machine learning approaches. The R^2^ statistic is used to determine the amount of variance in a response variable provided by a model. In other words, it expresses the model’s fit to the data quantitatively. A number around zero implies that fitting the mean is comparable to fitting the model, but a value near one shows that the data and model are nearly completely matched [42]. The subsections below discuss the machine learning techniques employed in this study. Moreover, all machine learning methods are validated using k-fold assessment, statistical checks, and error measures (root-mean-square error (RMSE) and mean absolute error (MAE)). Furthermore, sensitivity analysis is performed to determine the effect of each input variable on the predicted findings. The flow diagram in Figure 3 illustrates the research method used in this study.

#### 2.2.1. Gradient Boosting

**Figure 4:**
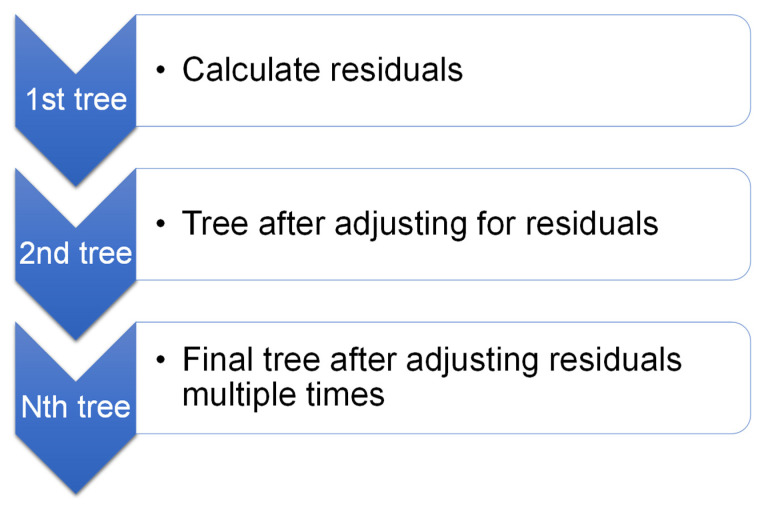
Schematic representation of the gradient boosting technique.

Friedman [43] presented gradient boosting as an ensemble strategy for classification and regression in 1999. Gradient boosting is only applicable to regression. As seen in Figure 4, the gradient boosting technique compares each iteration of the randomly chosen training set to the base model. A weak predictor is constructed using all of the training data. Then, the training data are predicted using a weak predictor. With the expected outcome, it is simple to calculate the residuals for each training instance. Gradient boosting for execution may be sped up and accuracy increased by randomly subsampling the training data, which also helps to prevent overfitting. The lower the training data percentage, the faster the regression, because the model must suit minor data every single iteration. Gradient boosting algorithms require tuning parameters, including n-trees and shrinkage rate, where n-trees is the number of trees to be generated; n-trees must not be kept too low, while the shrinkage factor—normally referred to as the learning rate employed to all trees in the development—should not be set too high [44].

#### 2.2.2. Random Forest

**Figure 5:**
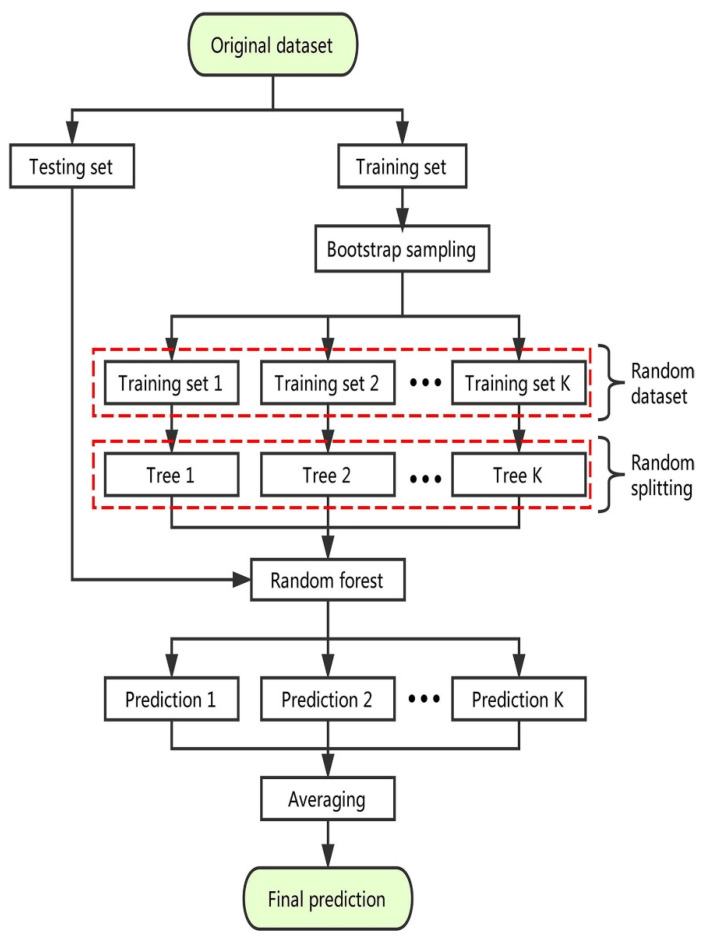
Schematic representation of the random forest technique [45].

Random forest are deployed by bagging decision trees using the random split choice technique [45]. The modeling procedure for the random forest approach is illustrated schematically in Figure 5. Each tree in the forest is generated by means of an arbitrarily selected training set, and each split inside a tree is constructed by means of an arbitrarily chosen subgroup of input factors, yielding a forest of trees [46]. This element of instability adds variation to the tree. The forest as a whole is composed completely of mature binary trees. The random forest technique has established itself as a highly effective tool for general-purpose classification and regression. When the number of variables surpasses the number of observations, the technique has proven improved precision by aggregating the predictions of several randomized decision trees. Additionally, it is adaptable to both large-scale and ad hoc learning tasks, yielding measures of varying degrees of importance [47].

## 3. Results and Analysis

### 3.1. Gradient Boosting Model

#### 3.1.1. Compressive Strength

**Figure 6:**
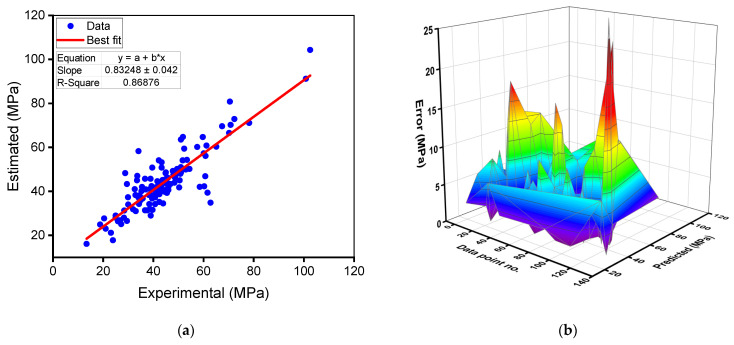
Gradient boosting model for compressive strength: (**a**) relationship between experimental and predicted results; (**b**) spreading of predicted and error values.

The results of the gradient boosting model for RAC’s compressive strength are shown in Figure 6a,b. Figure 6a depicts the relationships between the experimental and anticipated results. The gradient boosting approach yielded findings with a satisfactory level of accuracy and a lower distinction between the experimental and projected values. The R^2^ of 0.87 signifies that the gradient boosting model is reasonably precise at forecasting the compressive strength of RAC. The distribution of forecast and error values for the gradient boosting compressive strength model is presented in Figure 6b. The discrepancy between experimental and estimated values was found to be between 0.00 and 27.96 MPa (44.52% deviation), with an average of 4.78 MPa (11.67%). Additionally, the divergence from the experimental outcomes was less than 1 MPa for 27 mixes, between 1 and 3 MPa for 32 mixes, between 3 and 6 MPa for 32 mixes, between 6 and 10 MPa for 21 mixes, and greater than 10 MPa for 16 mixes. These deviations indicate that the gradient boosting model’s predicted results deviated less from the experimental results. As a result, the gradient boosting technique is quite accurate at predicting RAC’s compressive strength.

#### 3.1.2. Flexural Strength

**Figure 7:**
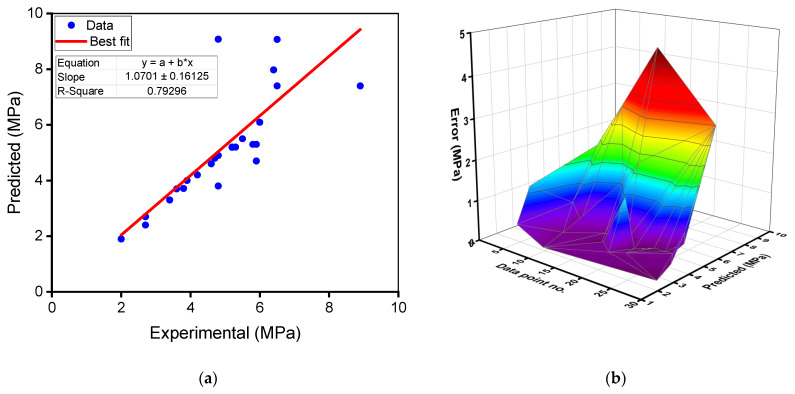
Gradient boosting model for flexural strength: (**a**) relationship between experimental and predicted results; (**b**) spreading of predicted and error values.

Figure 7a,b provides a comparison of the experimental and predicted outcomes of the gradient boosting model for the flexural strength of RAC. The correlation between experimental and estimated findings is exemplified in Figure 7a, where an R^2^ of 0.79 indicates that the gradient boosting model for the flexural strength is less specific than for the compressive strength estimation of RAC. This reduced R^2^ is due to the lower number of data points used for forecasting the flexural strength compared to the compressive strength. The distribution of estimated and error values for the gradient boosting flexural strength model is represented in Figure 7b. The difference between experimental and estimated values was discovered to be between 0.00 and 4.27 MPa (89.27% deviation), with an average of 5.86 MPa (11.44%). Furthermore, the difference from the experimental outcomes was less than 1 MPa for 22 mixes and greater than 1 MPa for 6 mixes. These deviation values suggest a moderate disparity between the gradient boosting model’s projected and experimental outcomes. As a result, the gradient boosting approach predicts RAC’s flexural strength less accurately compared to its precision in foretelling the compressive strength of RAC.

### 3.2. Random Forest Model

#### 3.2.1. Compressive Strength

**Figure 8:**
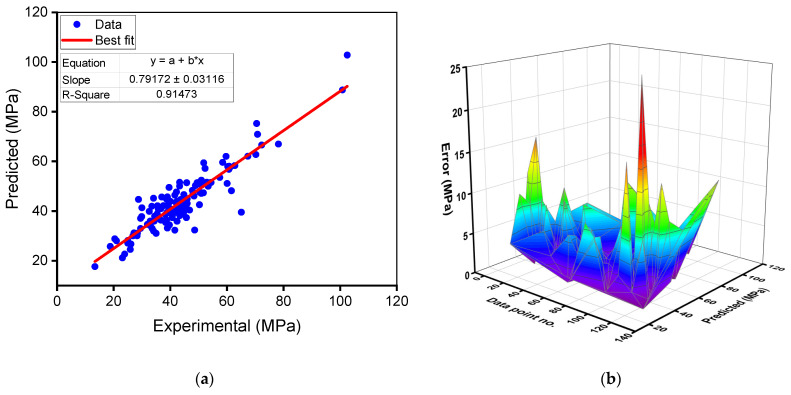
Random forest model for compressive strength: (**a**) relationship between experimental and predicted results; (**b**) spreading of predicted and error values.

The outcomes of the random forest model for the compressive strength of RAC are presented in Figure 8. In Figure 8a, an R^2^ value of 0.91 indicates that the random forest model outperforms the gradient boosting model in this study in terms of precision. The dispersion of projected and error values for the random forest compressive strength model is shown in Figure 8b. The variation (error) between experimental and estimated values was found to range between 0.07 and 25.57 MPa (39.28% variation), with an average of 4.19 MPa (10.50% variation). Furthermore, the difference from the experimental outcomes was less than 1 MPa for 18 mixes, between 1 and 3 MPa for 41 mixes, between 3 and 6 MPa for 39 mixes, between 6 and 10 MPa for 22 mixes, and larger than 10 MPa for only 8 mixes. These values show that the difference between experimental and expected outcomes is less compared to the gradient boosting model. As a result, the random forest approach is superior for assessing the compressive strength of RAC with the greatest precision.

#### 3.2.2. Flexural Strength

**Figure 9:**
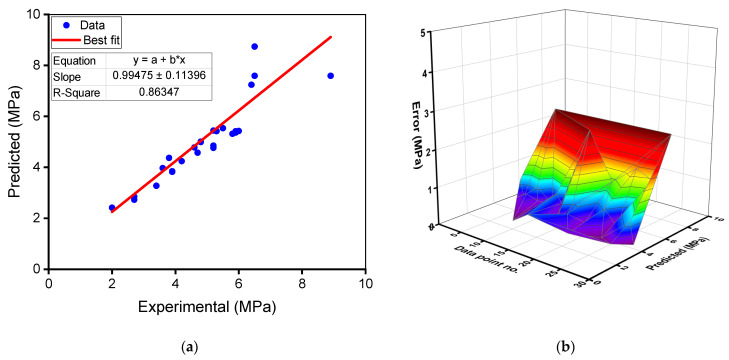
Random forest model for flexural strength: (**a**) relationship between experimental and predicted results; (**b**) spreading of predicted and error values.

The experimental and anticipated outcomes of the random forest model for the flexural strength of RAC are shown in Figure 9. Figure 9a represents the relationships between experimental and projected outcomes, with an R^2^ of 0.86 indicating that the random forest model for the flexural strength is less specific than the compressive strength prediction of RAC. This reduced R^2^ is because there are fewer data points used to forecast the flexural strength than the compressive strength. Figure 9b indicates the distribution of estimated and error values for the random forest flexural strength model. The discrepancy between experimental and estimated values ranged from 0.02 to 2.24 MPa (34.46 variances), with an average of 0.56 MPa (10.43% variance). Moreover, for 23 mixes, the variation from the experimental outcomes was less than 1 MPa, whereas it was greater than 1 MPa for only 5 mixes. These values indicate a lower difference between the random forest model’s predicted and experimental results. As a result, the random forest technique is more accurate in forecasting RAC’s flexural strength than the gradient boosting model.

### 3.3. Models’ Validation

**Figure 10:**
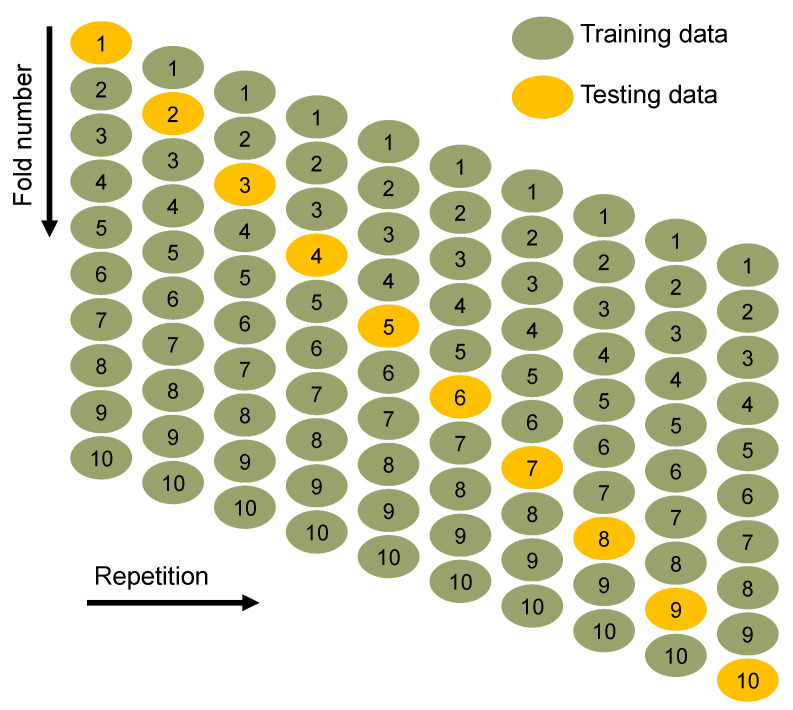
K-fold analysis procedure [50].

The machine learning methods were validated by employing k-fold and statistical methods. The k-fold technique, in which related data are randomly spread and separated into 10 groups, is widely used to determine a technique’s validity [48]. Nine groups are employed for training the model, and one group is used for validation, as shown in Figure 10. The model is more accurate when the errors (MAE and RMSE) are less and the R^2^ is high. In order to get a reasonable conclusion, the operation should be repeated 10 times. The model’s outstanding accuracy is due in large part to this enormous effort. In addition, both models were statistically tested based on errors (MAE and RMSE), as shown in Table 3. In comparison to the gradient boosting technique, this assessment also validated the random forest model’s superior accuracy due to reduced error readings. Equations (1) and (2), which were obtained from prior investigations [31,49], were used to determine the approaches’ prediction performance statistically.

(1) $$\mathrm{MAE}=\frac{1}{n}\sum_{i=1}^{n}\left|P_{i}-T_{i}\right|,$$

(2) $$\mathrm{RMSE}=\sqrt{\sum \frac{{\left(P_{i}-T_{i}\right)}^{2}}{n}},$$

where n = total number of data points, $T_{i}$ = experimental value, and $P_{i}$ = predicted value.

**Figure 11:**
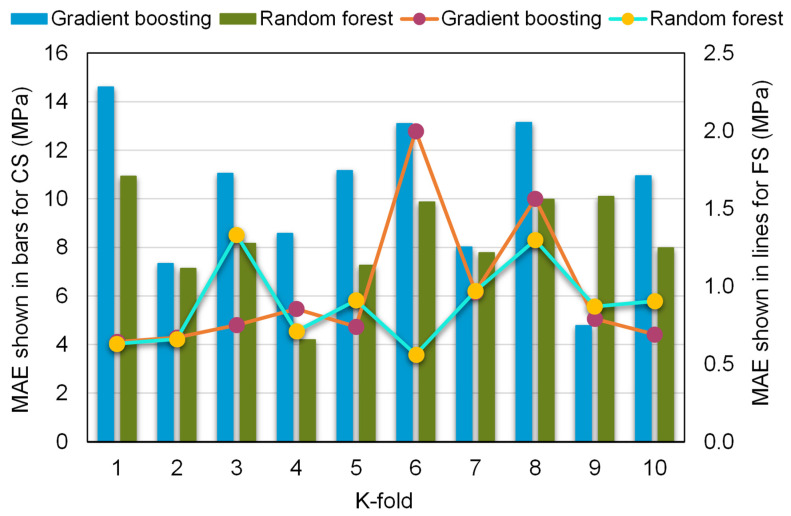
Mean absolute error distribution from k-fold analysis.

**Figure 12:**
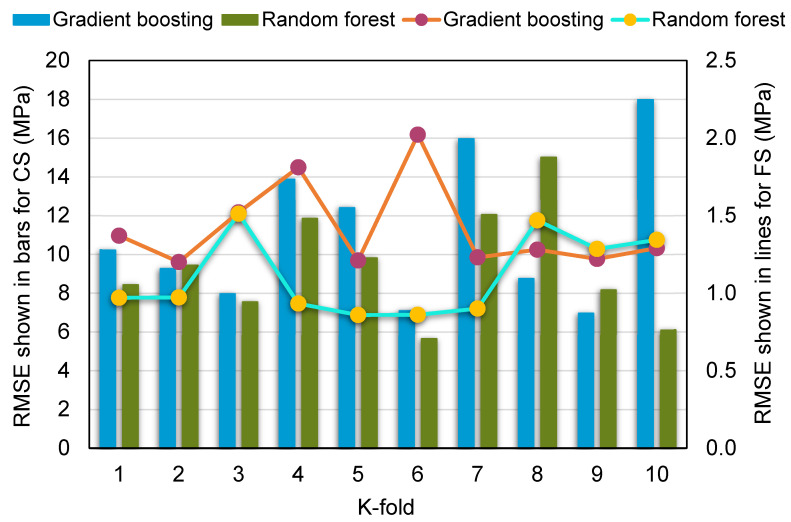
Root-mean-square error distribution from k-fold analysis.

**Figure 13:**
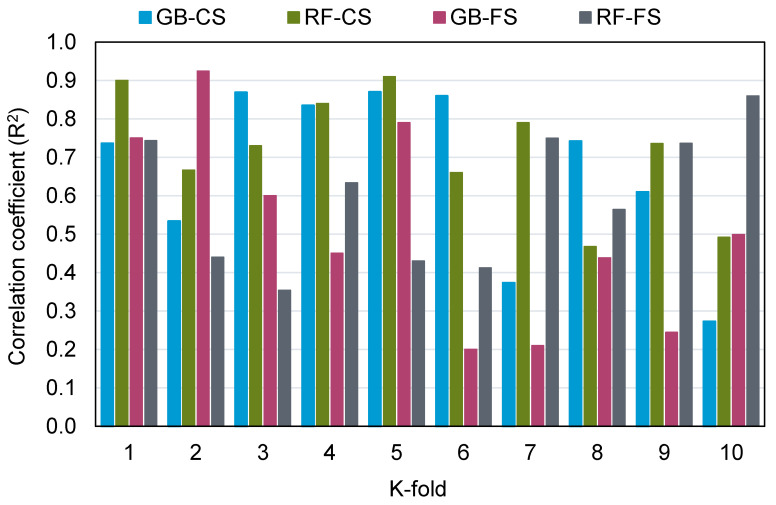
Correlation coefficient (R^2^) distribution from the k-fold analysis. GB: gradient boosting, RF: random forest, CS: compressive strength, FS: flexural strength.

MAE, RMSE, and R^2^ were measured to see how well the k-fold analysis was executed, and the results are shown in Table 4. Figure 11, Figure 12 and Figure 13 show a comparison of k-fold analysis for all of the machine learning techniques used. The MAE values for the gradient boosting compressive strength model ranged from 4.78 to 14.60 MPa, with an average of 10.27 MPa. In contrast, the MAE values for the random forest compressive strength model ranged from 4.19 to 10.92 MPa, with an average of 8.34 MPa. Likewise, the gradient boosting and random forest models for the compressive strength of RAC had average RMSE values of 11.05 and 9.41 MPa, respectively. When R^2^ values were evaluated, the average R^2^ values for the gradient boosting and random forest models were 0.67 and 0.72, respectively. When compared to the gradient boosting model, the random forest model—with smaller error values and greater R^2^ values—was more precise in projecting the compressive strength of RAC. A similar distribution of error and R^2^ values was discovered for the flexural strength of RAC for both the gradient boosting and random forest models, and this also validated the higher precision of the random forest model. Hence, the random forest model might be employed for the strength estimation of RAC in order to reduce the number of trials required for experimentation.

### 3.4. Sensitivity Analysis

**Figure 14:**
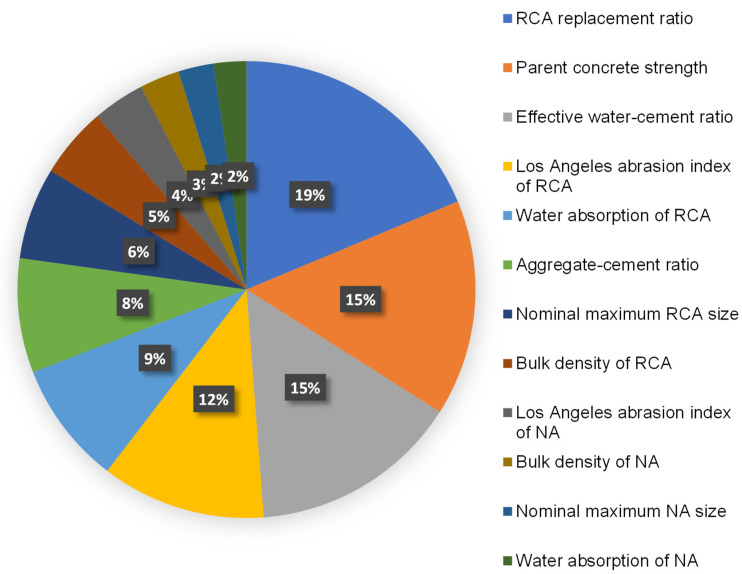
Input variables’ contribution to estimating the outcomes of models.

The purpose of this evaluation was to discover the impact of input factors on RAC’s compressive and flexural strength prediction. The anticipated result is considerably influenced by the input factors [51]. Figure 14 shows the influence of the input factors used in this research on the compressive strength evaluation of RAC. The analysis revealed that the RCA replacement ratio was the crucial element, accounting for 18.7% of the overall impact, followed by parent concrete strength at 15.3% and w_eff_/c at 14.8%. The contribution of the other input factors to the strength estimation of RAC was found to be lower, with the Los Angeles abrasion index of RCA, water absorption of RCA, a/c, nominal maximum RCA size, bulk density of RCA, Los Angeles abrasion index of natural aggregate, bulk density of the natural aggregate, nominal maximum natural aggregate size, and water absorption of the natural aggregate accounting for 11.6%, 8.7%, 8.1%, 6.5%, 5.0%, 3.7%, 2.8%, 2.5%, and 2.3%, respectively. Sensitivity analysis produced results associated with the quantity of input variables and the dataset used to build the machine learning models. The impact of an input factor on the method’s results was found using Equations (3) and (4).

(3) $$N_{i}=f_{max}\left(x_{i}\right)-f_{min}\left(x_{i}\right)$$

(4) $$S_{i}=\frac{N_{i}}{\sum_{j-i}^{n}N_{j}}.$$

where

$f_{max}\left(x_{i}\right)$ = highest estimated value on the $i^{th}$ result;
$f_{min}\left(x_{i}\right)$ = lowest estimated value on the $i^{th}$ result;
$S_{i}$ = attained impact percentage for a certain variable.

## 4. Discussions

**Figure 15:**
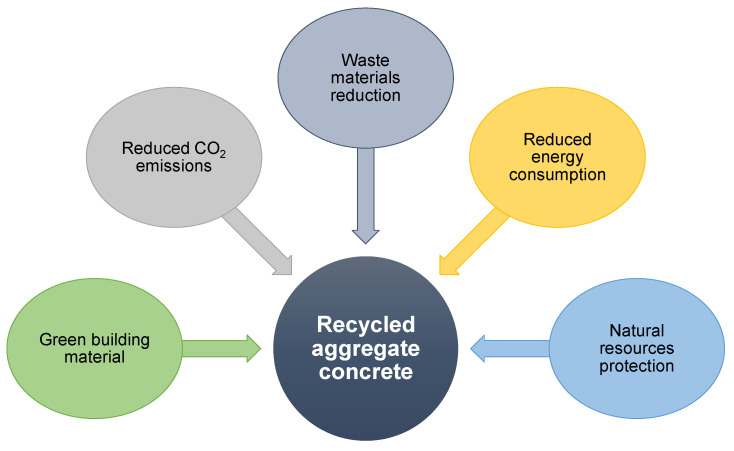
Benefits related to the adoption and application of recycled aggregate concrete.

The goal of this study was to add to the existing domain of research on the use of modern methods for evaluating the strength of RAC. This sort of exploration will benefit the building sector by allowing for the advancement of fast and cost-effective material property projection methods. Furthermore, by implementing these techniques to encourage environmentally friendly construction, the acceptance and usage of RAC in the building sector could be expedited. Figure 15 depicts the advantages of adopting RAC in the construction industry. Significant infrastructural renovation is required as a result of urbanization and industrialization, resulting in high volumes of construction and demolition waste. Therefore, desirable areas are turned into garbage ditches, land prices continue to rise, and trash dumping costs rise, with landfill space becoming increasingly rare. As a result, waste management has become of leading significance in emerging countries and is a global concern that demands long-term solutions. In addition, extracting and processing natural aggregates for concrete uses a lot of energy and produces a lot of CO_2_ [52]. Thus, using RAC in concrete production could result in lower energy consumption, resource conservation, building sustainability, cost savings, and a significant decrease in construction and demolition waste.

This research shows how machine learning methods may be used to forecast the compressive and flexural strength of RAC. The study employed two ensemble machine learning techniques—gradient boosting and random forest—to determine which technique is the most accurate predictor. The random forest model, with an R^2^ of 0.91 for compressive strength and 0.86 for flexural strength prediction, suggested a higher precision compared to the gradient boosting model, which produced R^2^ of 0.87 and 0.79 for compressive and flexural strength prediction, respectively. Furthermore, the accuracy of all machine learning methods was tested through the use of k-fold and statistical methods. The model is more precise if there are fewer error values in it. However, selecting and suggesting the best machine learning model for forecasting outcomes in a range of fields is difficult, because a model’s validity is highly dependent on the input factors and size of the dataset employed [53]. Ensemble machine learning techniques frequently take advantage of the weak learner by building 20 submodels that might be trained on data and altered to maximize the R^2^ value. The random forest model has also been found to be more exact in forecasting the strength of concrete by other researchers [54,55,56] in terms of R^2^ and error values. Farooq et al. [54] compared the functioning of random forest with that of the artificial neural network, gene expression programming, and decision tree methods, and found that the random forest model, with an R^2^ of 0.96, had a higher precision than the others. The reason for the higher accuracy of random forest is that it employs the bagging approach to combine all regression trees [57,58]. By minimizing the variation associated with prediction, bagging can increase prediction accuracy.

**Figure 16:**
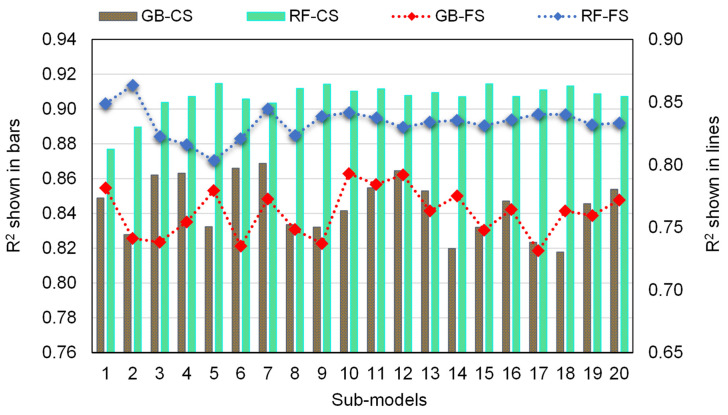
Correlation coefficients (R^2^) of submodels.

Figure 16 depicts the R^2^ value dispersion for the gradient boosting and random forest submodels. For gradient boosting compressive strength submodels, the lowest, average, and maximum R^2^ values were 0.818, 0.844, and 0.869, respectively. Additionally, the least, average, and highest R^2^ values for the gradient boosting flexural strength submodels were noted to be 0.731, 0.762, and 0.793, respectively. Similarly, for random forest compressive strength submodels, the lowest, average, and highest R^2^ values were 0.877, 0.907, and 0.915, respectively. Meanwhile, the least, average, and greatest R^2^ values for the random forest flexural strength submodels were identified to be 0.803, 0.834, and 0.863, respectively. These findings revealed that the random forest submodels had greater R^2^ values than the gradient boosting submodels, indicating that the random forest model was more precise in estimating RAC’s strength. A sensitivity analysis was also conducted to determine the effects of all inputs on the projected strength of RAC. The size of the dataset and the input parameters may have an impact on the model’s performance. The sensitivity analysis determined the contributions of each of the 12 input parameters to the expected output. The three most important input factors were discovered to be the RCA replacement ratio, parent concrete strength, and w_eff_/c.

## 5. Conclusions

This study aimed to employ two ensemble machine learning algorithms to anticipate the compressive and flexural strength of recycled aggregate concrete (RAC). Gradient boosting and random forest were chosen to achieve the study’s goals. The dataset containing the strength of RAC of 638 mixes was collected, of which all contained compressive strength results and 139 contained flexural strength results. Both gradient boosting and random forest models were employed to predict the compressive and flexural strength of RAC, and their accuracy was compared. The conclusions of this study are as follows:

1. The random forest model outperformed the gradient boosting model in estimating the compressive and flexural strength of RAC, with an R^2^ value of 0.91 for compressive strength and 0.86 for flexural strength prediction. However, the results of the gradient boosting model for the compressive strength estimation of RAC were also in the reasonable range, with an R^2^ of 0.87, but for the flexural strength estimation, the accuracy of the gradient boosting model was lower, with an R^2^ of 0.79. The lower R^2^ values for the flexural strength estimation in both models were because of the lower number of input data points. Hence, the random forest technique is suitable to be used for the strength prediction of RAC;
2. The analysis of predicted results indicated a lower variance from the experimental results for the random forest model compared to the gradient boosting model, which also validated the higher precision of the random forest model in predicting the strength of RAC;
3. K-fold and statistical evaluations further validated the model’s precision. These assessments also validated the higher precision of the random forest model due to the lower error values in comparison with the gradient boosting model;
4. Sensitivity analysis revealed that the RCA replacement ratio was the most important constituent affecting the model’s outcome, accounting for 18.7% of the total, followed by parent concrete strength at 15.3% and the effective water–cement ratio at 14.8%. However, the other input parameters had less contribution to the forecast of RAC’s compressive strength, with the Los Angeles abrasion index of RCA, water absorption of RCA, a/c, nominal maximum RCA size, bulk density of RCA, Los Angeles abrasion index of natural aggregate, bulk density of natural aggregate, nominal maximum natural aggregate size, and water absorption of the natural aggregate accounting for 11.6%, 8.7%, 8.1%, 6.5%, 5.0%, 3.7%, 2.8%, 2.5%, and 2.3%, respectively;
5. This sort of study will benefit the building sector by allowing for the advancement of rapid and cost-effective techniques for estimating the strength of materials. Furthermore, by encouraging computational techniques, the adoption and application of RAC in the building sector will be accelerated.

This study proposes that future studies should use experimental research, mixture proportions, field trials, and other numerical assessment methods to increase the amount of data points and findings (e.g., Monte Carlo simulation). Furthermore, to enhance the models’ responsiveness, environmental characteristics (e.g., elevated/low temperature and humidity) and a full description of the raw materials may be included as input variables.

## Figures and Tables

**Table 1:** Descriptive statistical values of input factors for the compressive strength dataset.

Parameter	RCA Replacement Ratio (%)	Parent Concrete Strength (MPa)	Bulk Density of NA (kg/m^3^)	Bulk Density of RCA (kg/m^3^)	Water Absorption of NA (%)	Water Absorption of RCA (%)	Aggregate–Cement Ratio (a/c)	Effective Water–Cement Ratio (w_eff_/c)	Nominal Maximum NA Size (mm)	Nominal Maximum RCA Size (mm)	Los Angeles Abrasion Index of NA	Los Angeles Abrasion Index of RCA
Mean	53.03	5.00	1538.47	1666.16	0.61	3.49	2.99	0.49	22.14	21.51	4.61	6.75
Range	100.00	100.00	2970.00	2880.00	3.00	11.90	6.50	0.87	38.00	32.00	32.00	42.00
Mode	100.00	0.00	0.00	0.00	0.00	0.00	3.10	0.50	20.00	20.00	0.00	0.00
Maximum	100.00	100.00	2970.00	2880.00	3.00	11.90	6.50	0.87	38.00	32.00	32.00	42.00
Minimum	0.00	0.00	0.00	0.00	0.00	0.00	0.00	0.00	0.00	0.00	0.00	0.00
Median	50.00	0.00	2570.00	2330.00	0.40	3.90	2.90	0.49	20.00	20.00	0.00	0.00
Standard Deviation	40.01	15.38	1315.12	1115.04	0.73	2.94	0.83	0.11	5.48	5.71	10.04	13.89
Sum	33,884	3193	983,081	1,064,677	391	2231	1913	312	14,149	13,747	2943	4312

**Table 2:** Descriptive statistical values of input factors for the flexural strength dataset.

Parameter	RCA Replacement Ratio (%)	Parent Concrete Strength (MPa)	Bulk Density of NA (kg/m^3^)	Bulk Density of RCA (kg/m^3^)	Water Absorption of NA (%)	Water Absorption of RCA (%)	Aggregate–Cement Ratio (a/c)	Effective Water–Cement Ratio (w_eff_/c)	Nominal Maximum NA Size (mm)	Nominal Maximum RCA Size (mm)	Los Angeles Abrasion of NA	Los Angeles Abrasion of RCA
Mean	50.74	4.32	1704.24	1823.81	0.70	4.15	3.05	0.52	19.40	19.23	9.32	12.82
Range	100.00	100.00	2820.00	2578.00	2.10	11.90	6.00	0.87	30.00	32.00	32.00	41.40
Mode	100.00	0.00	0.00	0.00	0.00	0.00	2.80	0.50	20.00	20.00	0.00	0.00
Maximum	100.00	100.00	2820.00	2578.00	2.10	11.90	6.00	0.87	30.00	32.00	32.00	41.40
Minimum	0.00	0.00	0.00	0.00	0.00	0.00	0.00	0.00	0.00	0.00	0.00	0.00
Median	50.00	0.00	2590.00	2336.00	0.50	4.70	2.90	0.50	20.00	20.00	0.00	0.00
Standard Error	3.42	1.50	108.88	85.16	0.06	0.24	0.07	0.01	0.34	0.38	1.05	1.36
Standard Deviation	40.30	17.65	1283.62	1004.03	0.70	2.81	0.81	0.14	4.00	4.49	12.33	15.99
Sum	7053.00	600.00	236,890.00	253,509.00	96.90	577.28	423.40	71.75	2696.00	2673.00	1294.90	1781.30

NA: natural aggregate, RCA: recycled concrete aggregate.

**Table 3:** Statistical measurements of the models for validation.

Model	Compressive Strength (MPa)		Flexural Strength (MPa)	
	MAE	RMSE	MAE	RMSE
Gradient Boosting	4.776	6.976	0.642	1.199
Random Forest	4.194	5.642	0.560	0.859

**Table 4:** Results of k-fold analysis.

K-Fold	Compressive Strength						Flexural Strength					
	Gradient Boosting			Random Forest			Gradient Boosting			Random Forest		
	MAE	RMSE	R^2^	MAE	RMSE	R^2^	MAE	RMSE	R^2^	MAE	RMSE	R^2^
1	14.60	10.23	0.74	10.92	8.44	0.90	0.64	1.37	0.75	0.63	0.97	0.74
2	7.33	9.28	0.53	7.13	9.45	0.67	0.67	1.20	0.92	0.66	0.97	0.44
3	11.04	7.98	0.87	8.16	7.56	0.73	0.75	1.52	0.60	1.33	1.51	0.35
4	8.57	13.86	0.84	4.19	11.87	0.84	0.85	1.81	0.45	0.71	0.93	0.63
5	11.16	12.42	0.87	7.25	9.83	0.91	0.74	1.21	0.79	0.91	0.86	0.43
6	13.10	7.10	0.86	9.87	5.64	0.66	2.00	2.02	0.20	0.56	0.86	0.41
7	8.01	15.95	0.37	7.78	12.06	0.79	0.96	1.23	0.21	0.97	0.90	0.75
8	13.14	8.76	0.74	9.98	15.00	0.47	1.56	1.28	0.44	1.30	1.47	0.56
9	4.78	6.98	0.61	10.09	8.18	0.74	0.79	1.22	0.24	0.87	1.28	0.74
10	10.94	17.97	0.27	7.98	6.10	0.49	0.69	1.29	0.50	0.90	1.34	0.86

## Data Availability

The data used in this research has been properly cited and reported in the main text.

## Supplementary Materials

The following are available online at https://www.mdpi.com/article/10.3390/ma15082823/s1, Table S1: Data used for modeling. References [59,60,61,62,63,64,65,66,67,68,69,70,71,72,73,74,75,76,77,78,79,80,81,82,83,84,85,86,87,88,89,90,91,92,93,94,95,96,97,98,99,100,101,102,103,104,105,106,107,108,109,110,111,112,113,114,115,116,117,118,119,120,121] are cited in the Supplementary Materials.
