# Correction: Yuan et al. Machine Learning Prediction Models to Evaluate the Strength of Recycled Aggregate Concrete. *Materials* 2022, *15*, 2823

**DOI:** 10.3390/ma18235288

**Published:** 2025-11-24

**Authors:** Xiongzhou Yuan, Yuze Tian, Waqas Ahmad, Ayaz Ahmad, Kseniia Iurevna Usanova, Abdeliazim Mustafa Mohamed, Rana Khallaf

**Affiliations:** 1 School of Traffic and Environment, Shenzhen Institute of Information Technology, Shenzhen 518172, China; 2 School of Civil Engineering, University of Science and Technology Liaoning, Anshan 114051, China; 3 Department of Civil Engineering, COMSATS University Islamabad, Abbottabad 22060, Pakistan; 4 MaREI Centre, Ryan Institute, School of Engineering, College of Science and Engineering, National University of Ireland, H91 TK33 Galway, Ireland; 5 Peter the Great St. Petersburg Polytechnic University, 195291 St. Petersburg, Russia; 6 Department of Civil Engineering, College of Engineering in Al-Kharj, Prince Sattam Bin Abdulaziz University, Al-Kharj 11942, Saudi Arabia; 7 Building & Construction Technology Department, Bayan College of Science and Technology, Khartoum 11115, Sudan; 8 Structural Engineering and Construction Management, Faculty of Engineering and Technology, Future University in Egypt, New Cairo 11845, Egypt

There is a statement missing in the original publication [1] regarding data in Tables 1 and 2—“The entries in Tables 1 and 2 with 0 (zero) do not represent actual physical measurements. Instead, these zero values indicate unavailable data from the original studies”.

A correction has been made to Section 2.1. Data Retrieval and Analysis, paragraph 2:

In addition, the compressive and flexural strength were chosen as the output variables. The quantity of input variables and the dataset have a substantial impact on a machine learning method’s result [37–39]. In the present study, 638 data points (mixes) were employed to run machine learning methods for compressive strength prediction, and 139 data points (mixes) were used for flexural strength prediction. Tables 1 and 2 summarize the descriptive statistic evaluation of each input variable for compressive and flexural strength prediction, respectively. The mode, median, and mean exemplify basic propensity, while the standard deviation, minimum, and maximum denote variability. The entries in Tables 1 and 2 with 0 (zero) do not represent actual physical measurements. Instead, these zero values indicate unavailable data from the original studies. The relative frequency dispersal of input factors employed to forecast the compressive and flexural strength is depicted in Figures 1 and 2, respectively. This represents the overall number of readings linked to each input parameter.

The authors state that the scientific conclusions are unaffected. This correction was approved by the Academic Editor. The original publication has also been updated.
