# Supplementary material for: Machine Learning Prediction Models to Evaluate the Strength of Recycled Aggregate Concrete
Source: Materials (Basel). 2022 Apr 12;15(8):2823. doi: 10.3390/ma15082823 (PMC9025364; doi:10.3390/ma15082823)
Supplement: Supplementary file 1 [file materials-15-02823-s001.zip › materials-1645501-supplementary.pdf]

materials

Article

Machine Learning Prediction Models to Evaluate the Strength of Recycled Aggregate Concrete

Xiongzhou Yuan [1], Yuze Tian [2,*], Waqas Ahmad [3,*], Ayaz Ahmad [3,4], Kseniia Iurevna Usanova [5], Abdeliazim Mustafa Mohamed [6,7] and Rana Khallaf [8]

[1] School of Traffic and Environment, Shenzhen Institute of Information Technology, ShenZhen 518172, China; couscous_yuan@sina.com
[2] School of Civil Engineering, University of Science and Technology Liaoning, AnShan 114051, China
[3] Department of Civil Engineering, COMSATS University Islamabad, Abbottabad 22060, Pakistan; ayazahmad@cuiatd.edu.pk
[4] MaREI Centre, Ryan Institute and School of Engineering, College of Science and Engineering, National University of Ireland, Galway, Ireland
[5] Peter the Great St. Petersburg Polytechnic University, 195291 St. Petersburg, Russia; usanova_kyu@spbstu.ru
[6] Department of Civil Engineering, College of Engineering in Al-Kharj, Prince Sattam bin Abdulaziz University, Al-Kharj 11942, Saudi Arabia; a.bilal@psau.edu.sa
[7] Building & Construction Technology Department, Bayan College of Science and Technology, Khartoum 11115, Sudan
[8] Structural Engineering and Construction Management, Faculty of Engineering and Technology, Future University in Egypt, New Cairo 11845, Egypt; rana.khallaf@fue.edu.eg
* Correspondence: 319933700033@ustl.edu.cn (Y.T.); waqasahmad@cuiatd.edu.pk (W.A.)

Citation: Yuan, X.; Tian, Y.; Ahmad, W.; Ahmad, A.; Usanova, K.I.; Mohamed, A.M.; Khallaf, R. Machine Learning Prediction Models to Evaluate the Strength of Recycled Aggregate Concrete. *Materials* **2022**, *15*, 2823. https://doi.org/10.3390/ma15082823

Academic Editor: Krzysztof Schabowicz

Received: 4 March 2022
Accepted: 27 March 2022
Published: 12 April 2022

Publisher's Note: MDPI stays neutral with regard to jurisdictional claims in published maps and institutional affiliations.

Copyright: © 2022 by the authors. Licensee MDPI, Basel, Switzerland. This article is an open access article distributed under the terms and conditions of the Creative Commons Attribution (CC BY) license (https://creativecommons.org/licenses/by/4.0/).

Table S1. Data used for modeling.

Ref.	Effective water-cement ratio (w_{eff}/c)	Aggregate-cement ratio (a/c)	RCA replacement ration (RCA %)	Parent concrete strength (MPa)	Nominal Maximum RCA size (mm)	Nominal Maximum NA size (mm)	Bulk density of RCA (kg/m³)	Bulk density of NA (kg/m³)	Water absorption of RCA (%)	Water absorption of NA (%)	Los Angeles abrasion of RCA	Los Angeles abrasion of NA	Compressive strength (MPa)	Flexural strength (MPa)
[1]	0.5	2.6	0	0	20	20	0	0	0	0	0	0	42.8	0
	0.5	2.5	20	0	20	20	0	0	0	0	0	0	42.7	0
	0.5	2.5	50	0	20	20	0	0	0	0	0	0	41.3	0
	0.5	2.3	100	0	20	20	0	0	0	0	0	0	41.8	0
[2]	0.45	3.3	0	0	20	30	0	2610	0	2.5	0	0	51.2	5.2
	0.45	3.3	30	0	20	30	2400	2610	4.9	2.5	0	0	50.6	5.2
	0.45	3.3	50	0	20	30	2400	2610	4.9	2.5	0	0	50.8	4.9
	0.45	3.3	100	0	20	30	2400	0	4.9	0	0	0	50.2	5
	0.39	2.6	0	0	20	30	0	2610	0	2.5	0	0	60.3	6
	0.39	2.6	30	0	20	30	2400	2610	4.9	2.5	0	0	60.8	6.1
	0.39	2.6	50	0	20	30	2400	2610	4.9	2.5	0	0	61.2	6.1
	0.39	2.6	100	0	20	30	2400	0	4.9	0	0	0	60.2	6
	0.29	2.2	0	0	20	30	0	2610	0	2.5	0	0	70.5	7
	0.29	2.2	30	0	20	30	2400	2610	4.9	2.5	0	0	70.2	6.9
	0.29	2.2	50	0	20	30	2400	2610	4.9	2.5	0	0	70.8	7
	0.29	2.2	100	0	20	30	2400	0	4.9	0	0	0	70	7.2
[3]	0.36	2.4	0	41.6	16	16	0	0	0	0	0	0	48.4	0
	0.36	2.3	100	41.6	16	16	0	0	0	0	0	0	44.5	0
	0.36	2.2	100	41.6	16	16	0	0	0	0	0	0	38.7	0
	0.36	2.4	0	50.6	16	16	0	0	0	0	0	0	48.9	0
	0.36	2.3	100	50.6	16	16	0	0	0	0	0	0	46.1	0
	0.36	2.2	100	50.6	16	16	0	0	0	0	0	0	42.4	0
	0.36	2.4	0	63.2	16	16	0	0	0	0	0	0	48.9	0
	0.36	2.3	100	63.2	16	16	0	0	0	0	0	0	52.5	0
	0.36	2.2	100	63.2	16	16	0	0	0	0	0	0	50.7	0
	0.36	2.4	0	35.6	16	16	0	0	0	0	0	0	48.9	0
	0.36	2.3	100	35.6	16	16	0	0	0	0	0	0	45.2	0

	0.36	2.2	100	35.6	16	16	0	0	0	0	0	0	42	0
	0.36	2.4	0	66	16	16	0	0	0	0	0	0	48.9	0
	0.36	2.3	100	66	16	16	0	0	0	0	0	0	49.6	0
	0.36	2.2	100	66	16	16	0	0	0	0	0	0	45.1	0
	0.36	2.7	0	72.3	16	16	0	0	0	0	0	0	52.3	0
	0.36	2.4	100	72.3	16	16	0	0	0	0	0	0	54.4	0
	0.36	2.3	100	72.3	16	16	0	0	0	0	0	0	48.2	0
[4]	0.47	2.5	0	38.4	20	20	0	2590	0	0.9	0	0	39	0
	0.47	2.5	15	38.4	20	20	2410	2590	5.8	0.9	0	0	38.1	0
	0.45	2.5	30	38.4	20	20	2410	2590	5.8	0.9	0	0	37	0
	0.42	2.4	60	38.4	20	20	2410	2590	5.8	0.9	0	0	35.8	0
	0.38	2.3	100	38.4	20	20	2410	0	5.8	0	0	0	34.5	0
[5]	0.6	4.6	0	0	15	20	0	2670	0	0.5	0	0	43.5	0
	0.6	4.1	100	0	15	20	2450	0	5.6	0	0	0	38.2	0
	0.45	3.3	0	0	15	20	0	2670	0	0.5	0	0	61.7	0
	0.45	2.9	100	0	15	20	2450	0	5.6	0	0	0	52.8	0
	0.35	2.6	0	0	15	20	0	2670	0	0.5	0	0	74.4	0
	0.35	2.3	100	0	15	20	2450	0	5.6	0	0	0	62.8	0
	0.45	3.2	25	0	15	20	0	2670	0	0.5	0	0	60.7	0
	0.45	3.1	50	0	15	20	2450	0	5.6	0	0	0	59.4	0
[6]	0.57	3.1	0	0	20	25	0	2620	0	1.3	0	0	48.3	0
	0.57	3.1	20	0	20	25	2330	2620	6.3	1.3	0	0	44.9	0
	0.57	3.1	50	0	20	32	2330	2620	6.3	1.3	0	0	44.7	0
	0.57	3	100	0	20	32	2330	0	6.3	0	0	0	46.8	0
	0.57	3	0	0	20	32	0	2620	0	1.3	0	0	40.2	0
	0.57	3.1	20	0	20	32	2330	2620	6.3	1.3	0	0	43.2	0
	0.57	2.9	50	0	20	32	2330	2620	6.3	1.3	0	0	39.7	0
	0.57	2.9	100	0	20	32	2330	0	6.3	0	0	0	43.3	0
	0.57	3	0	0	20	20	0	2620	0	1.3	0	0	46	0
	0.57	2.8	20	0	20	20	2330	2620	6.3	1.3	0	0	43	0
	0.57	2.7	50	0	20	20	2330	2620	6.3	1.3	0	0	38.1	0
	0.57	2.9	100	0	20	20	2330	0	6.3	0	0	0	39.1	0
[7]	0.5	2.4	100	0	25	20	0	0	0	0	0	0	30.2	0
	0.5	2.3	100	0	25	20	0	0	0	0	0	0	36.2	0
	0.7	3.3	100	0	25	20	0	0	0	0	0	0	27.7	0

	0.7	3.2	100	0	25	20	0	0	0	0	0	0	20.4	0
[8]	0.43	3	0	0	32	19	0	2820	0	0.4	0	0	35.9	0
	0.43	2.9	33	0	32	19	2520	2820	9.3	0.4	0	0	34.1	0
	0.43	2.8	53	0	32	19	2520	2820	9.3	0.4	0	0	29.6	0
	0.43	2.8	72	0	32	19	2520	2820	9.3	0.4	0	0	30.3	0
	0.43	2.7	100	0	32	19	2520	0	9.3	0	0	0	26.7	0
[9]	0.42	3	0	0	32	32	0	2786	0	0.3	0	0	36.8	5
	0.37	2.9	30	0	32	32	2442	2786	6	0.3	0	0	37.2	5
	0.34	2.8	50	0	32	32	2442	2786	6	0.3	0	0	37.8	5.2
	0.38	2	70	0	32	32	2442	2786	6	0.3	0	0	36.7	5.1
	0.27	2.7	100	0	32	32	2442	0	6	0	0	0	35.2	4.9
[10]	0.55	4	0	0	25	19	0	2670	0	0.9	0	0	42	0
	0.55	3.9	25	0	25	19	2430	2670	4.4	0.9	0	0	42	0
	0.52	3.6	50	0	25	19	2430	2670	4.4	0.9	0	0	41	0
	0.5	3.5	100	0	25	19	2430	0	4.4	0	0	0	40	0
[11]	0.55	4	0	0	25	19	0	2670	0	0.9	0	0	35.5	0
	0.55	3.9	25	0	25	19	2430	2670	4.5	0.9	0	0	38.8	0
	0.52	3.6	50	0	25	19	2430	2670	4.5	0.9	0	0	39.4	0
	0.5	3.5	100	0	25	19	2430	0	4.5	0	0	0	38.3	0
[12]	0.41	3.1	0	0	20	25	0	2564	0	0.8	0	0	59.4	0
	0.42	3.2	10	0	20	25	2165	2564	6.8	0.8	0	0	62.2	0
	0.43	3.4	20	0	20	25	2165	2564	6.8	0.8	0	0	58.4	0
	0.44	3.5	30	0	20	25	2165	2564	6.8	0.8	0	0	61.3	0
	0.45	3.7	50	0	20	25	2165	2564	6.8	0.8	0	0	60.8	0
	0.45	4.4	100	0	20	25	2165	0	6.8	0	0	0	61	0
[13]	0.51	2.6	0	0	20	20	0	2620	0	1.1	0	0	48.6	0
	0.49	2.5	20	0	20	20	2570	2620	3.5	1.1	0	0	45.3	0
	0.48	2.5	50	0	20	20	2570	2620	3.5	1.1	0	0	42.5	0
	0.46	2.5	80	0	20	20	2570	2620	3.5	1.1	0	0	39.2	0
	0.45	2.5	100	0	20	20	2570	0	3.5	0	0	0	37.1	0
[14]	0.49	4.7	0	0	16	20	2270	0	0	0	0	0	37.7	0
	0.49	3.9	100	0	16	20	2270	0	0	0	0	0	34.6	0
	0.36	2.4	0	0	16	20	2270	0	0	0	0	0	57.9	0
	0.36	2.2	100	0	16	20	2270	0	0	0	0	0	56.4	0
	0.49	3.7	0	0	16	25	2780	0	0	0	0	0	39.8	0

	0.49	4.4	100	0	16	25	2780	0	0	0	0	0	40.1	0
	0.36	2.4	0	0	16	25	2780	0	0	0	0	0	58.3	0
	0.36	2.3	100	0	16	25	2780	0	0	0	0	0	60.2	0
	0.49	5.1	0	0	16	16	2565	0	0	0	0	0	40.1	0
	0.49	4.2	100	0	16	16	2565	0	0	0	0	0	35.3	0
	0.36	2.7	0	0	16	16	2565	0	0	0	0	0	61.8	0
	0.36	2.4	100	0	16	16	2565	0	0	0	0	0	57.5	0
[15]	0.47	3.3	0	0	32	22	0	2788	0	0.3	0	0	31.2	4.6
	0.41	3.3	30	0	32	22	2449	2788	6	0.3	0	0	31	4.6
	0.38	3.2	50	0	32	22	2449	2788	6	0.3	0	0	29.3	4.4
	0.36	3.1	70	0	32	22	2449	2788	6	0.3	0	0	28.4	4.4
	0.32	3	100	0	32	22	2449	0	6	0	0	0	27.2	4.2
[16]	0.45	2.8	0	0	20	19	0	2620	0	1.1	0	0	66.8	0
	0.45	2.8	20	0	20	19	2570	2620	3.5	1.1	0	0	62.4	0
	0.45	2.7	50	0	20	19	2570	2620	3.5	1.1	0	0	55.8	0
	0.45	2.7	100	0	20	32	2570	0	3.5	0	0	0	42	0
	0.55	2.6	0	0	20	32	0	2620	0	1.1	0	0	48.6	0
	0.55	2.5	20	0	20	32	2570	2620	3.5	1.1	0	0	45.3	0
	0.55	2.5	50	0	20	32	2570	2620	3.5	1.1	0	0	42.5	0
	0.55	2.5	100	0	20	32	2570	0	3.5	0	0	0	38.1	0
[17]	0.65	3.1	0	0	19	25	0	2860	0	0.7	0	0	21.8	0
	0.65	3.1	100	0	19	25	2390	0	4.4	0	0	0	22.1	0
	0.5	2.9	0	0	19	25	0	2860	0	0.7	0	0	26.7	0
	0.5	2.9	100	0	19	25	2390	0	4.4	0	0	0	25.1	0
	0.48	2.8	0	0	19	25	0	2860	0	0.7	0	0	28.9	0
	0.48	2.8	100	0	19	25	2390	0	4.4	0	0	0	27.2	0
	0.43	2.6	0	0	19	25	0	2860	0	0.7	0	0	31.1	0
	0.43	2.6	100	0	19	25	2390	0	4.4	0	0	0	28.7	0
	0.4	2.4	0	0	19	19	0	2860	0	0.7	0	0	33.7	0
	0.4	2.4	100	0	19	19	2390	0	4.4	0	0	0	29.5	0
[18]	0.54	3.1	0	0	32	25	0	2840	0	0.4	0	0	26.8	0
	0.35	3.1	100	0	32	25	2512	0	6.3	0	0	0	24.6	0
	0.49	3.1	100	0	32	25	2670	0	1.8	0	0	0	26.9	0
	0.46	2.7	0	0	32	25	0	2840	0	0.4	0	0	34.3	0
	0.31	2.7	100	0	32	32	2512	0	6.3	0	0	0	30.2	0

	0.43	2.7	100	0	32	32	2670	0	1.8	0	0	0	34.2	0
	0.42	2.4	0	0	32	32	0	2840	0	0.4	0	0	38.6	0
	0.28	2.4	100	0	32	32	2512	0	6.3	0	0	0	35.5	0
	0.39	2.4	100	0	32	32	2670	0	1.8	0	0	0	38.4	0
[19]	0.7	4.1	0	0	30	10	0	2700	0	0.5	0	28	18.1	0
	0.67	3.9	100	0	30	10	2520	0	3.8	0	34	0	18	0
	0.67	3.9	100	0	30	10	2510	0	3.9	0	39	0	15.4	0
	0.35	3.1	0	0	30	10	0	2700	0	0.5	0	28	37.5	0
	0.35	4.3	100	0	30	10	2520	0	3.8	0	34	0	36.4	0
	0.36	2.1	100	0	30	10	2510	0	3.9	0	39	0	35.7	0
	0.34	2.1	0	0	30	19	0	2700	0	0.5	0	28	48.4	0
	0.34	1.9	100	0	30	19	2520	0	3.8	0	34	0	44.4	0
	0.34	2.2	100	0	30	30	2510	0	3.9	0	39	0	43.8	0
[20]	0.47	3.3	0	0	32	22	0	2788	0	0.3	0	0	31.2	4.6
	0.41	3.3	30	0	32	22	2449	2788	6	0.3	0	0	31	4.6
	0.38	3.2	50	0	32	22	2449	2788	6	0.3	0	0	29.3	4.4
	0.36	3.1	70	0	32	22	2449	2788	6	0.3	0	0	28.4	4.4
	0.32	3	100	0	32	25	2449	0	6	0	0	0	27.2	4.2
[21]	0.55	2.6	0	0	20	20	0	2620	0	1.1	0	0	48.6	0
	0.55	2.6	20	0	20	20	2580	2620	3.5	1.1	0	0	45.3	0
	0.55	2.5	50	0	20	20	2580	2620	3.5	1.1	0	0	42.5	0
	0.55	2.5	100	0	20	20	2580	0	3.5	0	0	0	38.1	0
	0.5	2.6	0	0	20	20	0	2620	0	1.1	0	0	54.1	0
	0.5	2.6	20	0	20	20	2580	2620	3.5	1.1	0	0	51.7	0
	0.5	2.6	50	0	20	20	2580	2620	3.5	1.1	0	0	47.1	0
	0.5	2.6	100	0	20	20	2580	0	3.5	0	0	0	43.4	0
	0.45	2.8	0	0	20	20	0	2620	0	1.1	0	0	66.8	0
	0.45	2.8	20	0	20	20	2580	2620	3.5	1.1	0	0	62.4	0
	0.45	2.7	50	0	20	20	2580	2620	3.5	1.1	0	0	56.8	0
	0.45	2.5	100	0	20	20	2580	0	3.5	0	0	0	52.1	0
	0.4	2.9	0	0	20	20	0	2620	0	1.1	0	0	72.3	0
	0.4	2.8	20	0	20	20	2580	2620	3.5	1.1	0	0	69.6	0
	0.4	2.8	50	0	20	20	2580	2620	3.5	1.1	0	0	65.3	0
	0.4	2.8	100	0	20	20	2580	0	3.5	0	0	0	58.5	0
[22]	0.5	2.9	0	0	25	20	0	2600	0	1.4	0	0	39.5	4

	0.5	2.9	30	0	25	20	2530	2600	1.9	1.4	0	0	36.7	4
	0.5	2.9	50	0	25	20	2530	2600	1.9	1.4	0	0	38	3.7
	0.5	2.8	100	0	25	32	2530	0	1.9	0	0	0	36	3.5
	0.5	2.8	30	0	25	32	0	2600	0	1.4	0	0	32.6	3.6
	0.5	2.8	50	0	25	32	2400	2600	6.2	1.4	0	0	30.4	3.4
	0.5	2.7	100	0	25	32	2400	0	6.2	0	0	0	29.5	3.2
[23]	0.58	3.2	0	0	32	32	0	2970	0	0.8	0	0	44.6	0
	0.52	3.2	50	0	32	22	2720	2970	4.8	0.8	0	0	41.4	0
	0.45	3.2	100	0	32	22	2720	0	4.8	0	0	0	40.7	0
	0.52	3.2	50	0	32	22	2650	2970	4.6	0.8	0	0	38.3	0
	0.46	3.2	100	0	32	22	2650	0	4.6	0	0	0	36.6	0
	0.52	3.2	50	0	32	22	2880	2970	4.4	0.8	0	0	41.2	0
	0.47	3.2	100	0	32	22	2880	0	4.4	0	0	0	40.3	0
[24]	0.41	2.6	0	0	20	20	0	2647	0	1	0	27.8	42.3	0
	0.39	2.5	20	0	20	20	2338	2647	5.2	1	40.2	27.8	47.4	0
	0.36	2.5	50	0	20	20	2338	2647	5.2	1	40.2	27.8	47.3	0
	0.32	2.3	100	0	20	20	2338	0	5.2	0	40.2	0	54.8	0
[25]	0.52	2.9	0	0	22	20	0	0	0	0	0	0	48	0
	0.52	2.9	10	0	22	20	0	0	0	0	0	0	46.9	0
	0.52	2.8	20	0	22	20	0	0	0	0	0	0	47.7	0
	0.52	2.8	30	0	22	20	0	0	0	0	0	0	50.8	0
	0.52	2.8	40	0	22	20	0	0	0	0	0	0	48	0
	0.52	2.8	50	0	22	25	0	0	0	0	0	0	49.5	0
	0.52	2.7	100	0	22	25	0	0	0	0	0	0	50.3	0
	0.54	3.2	0	0	22	25	0	0	0	0	0	0	23.5	3.1
	0.54	3.1	25	0	22	25	0	0	0	0	0	0	21.6	2.9
	0.54	3.1	100	0	22	20	0	0	0	0	0	0	20.5	3.1
[26]	0.76	4.5	100	0	30	32	0	0	0	0	0	0	21.1	0
	0.76	4.5	100	0	30	32	0	0	0	0	0	0	22	0
	0.76	4.5	100	0	30	32	0	0	0	0	0	0	23.1	0
	0.76	4.5	100	0	30	32	0	0	0	0	0	0	23.5	0
	0.76	4.5	100	0	30	32	0	0	0	0	0	0	20.4	0
	0.76	4.5	100	0	30	32	0	0	0	0	0	0	18.9	0
	0.76	4.5	100	0	30	32	0	0	0	0	0	0	21.2	0
	0.66	3.9	100	0	30	32	0	0	0	0	0	0	25.7	0

	0.66	3.9	100	0	30	32	0	0	0	0	0	0	28	0
	0.66	3.9	100	0	30	32	0	0	0	0	0	0	25.1	0
	0.66	3.9	100	0	30	32	0	0	0	0	0	0	27.5	0
	0.66	3.9	100	0	30	32	0	0	0	0	0	0	26.1	0
	0.66	3.9	100	0	30	32	0	0	0	0	0	0	27.4	0
	0.66	3.9	100	0	30	32	0	0	0	0	0	0	27.7	0
	0.66	3.9	100	0	30	32	0	0	0	0	0	0	25	0
	0.57	3.3	100	0	30	32	0	0	0	0	0	0	30.5	0
	0.57	3.3	100	0	30	30	0	0	0	0	0	0	32.7	0
	0.57	3.3	100	0	30	30	0	0	0	0	0	0	32.8	0
	0.57	3.3	100	0	30	30	0	0	0	0	0	0	33.1	0
	0.48	2.7	100	0	30	30	0	0	0	0	0	0	35.3	0
	0.48	2.7	100	0	30	30	0	0	0	0	0	0	35.2	0
	0.48	2.7	100	0	30	30	0	0	0	0	0	0	32.5	0
	0.48	2.7	100	0	30	30	0	0	0	0	0	0	33.8	0
	0.41	2.2	100	0	30	30	0	0	0	0	0	0	41.9	0
	0.41	2.2	100	0	30	30	0	0	0	0	0	0	38.4	0
	0.41	2.2	100	0	30	30	0	0	0	0	0	0	38.7	0
	0.41	2.2	100	0	30	30	0	0	0	0	0	0	41.2	0
[27]	0.54	3.1	0	0	32	32	0	2840	0	0.4	0	0	26.8	4.2
	0.35	3.1	100	0	32	32	2512	0	6.3	0	0	0	24.6	4.2
	0.49	3.1	100	0	32	32	2670	0	1.8	0	0	0	26.9	4.2
	0.46	3.3	0	0	32	32	0	2840	0	0.4	0	0	34.3	4.8
	0.27	3.3	100	0	32	32	2512	0	6.3	0	0	0	30.2	4.7
	0.41	3.3	100	0	32	32	2670	0	1.8	0	0	0	34.2	4.8
	0.42	3	0	0	32	32	0	2840	0	0.4	0	0	38.6	5.1
	0.24	3	100	0	32	32	2512	0	6.3	0	0	0	35.5	5
	0.38	3	100	0	32	32	2670	0	1.8	0	0	0	38.4	5.1
[28]	0.4	3.1	50	0	12	30	2420	2570	6.8	3	0	0	43.3	0
	0.45	3.1	50	0	12	30	2400	2570	6.8	3	0	0	39.6	0
	0.5	3.2	50	0	12	30	2400	2570	6.8	3	0	0	38.1	0
	0.55	3.2	50	0	12	30	2400	2570	6.8	3	0	0	34.5	0
	0.6	3.3	50	0	12	30	2400	2570	6.8	3	0	0	31.6	0
	0.4	3.1	50	0	22	30	2420	2570	8.8	3	0	0	46.1	0
	0.45	3.1	50	0	22	30	2420	2570	8.8	3	0	0	45.8	0

	0.5	3.2	50	0	22	30	2420	2570	8.8	3	0	0	39.9	0
	0.55	3.3	50	0	22	20	2420	2570	8.8	3	0	0	36.3	0
	0.6	3.3	50	0	22	20	2420	2570	8.8	3	0	0	34.7	0
[29]	0.5	3.5	0	0	20	20	0	2870	0	0	0	0	28.3	4.9
	0.5	3.5	20	0	20	20	2400	2870	0	0	0	0	27.2	3.9
	0.5	3.5	40	0	20	20	2400	2870	0	0	0	0	26.5	2.9
	0.5	3.5	60	0	20	20	2400	2870	0	0	0	0	25.4	2.7
	0.5	3.5	80	0	20	20	2400	2870	0	0	0	0	25.1	2.6
	0.5	3.5	100	0	20	20	2400	0	0	0	0	0	20.4	2.4
	0.5	3.8	20	0	20	20	2630	2870	0	0	0	0	26.4	4.4
	0.5	4.1	40	0	20	20	2630	2870	0	0	0	0	25.9	4.1
	0.5	4.5	60	0	20	20	2630	2870	0	0	0	0	23.5	3.8
	0.5	4.8	80	0	20	20	2630	2870	0	0	0	0	15.4	3.4
[30]	0.51	3.6	0	0	32	20	0	2671	0	0.3	0	29.2	43.4	5.4
	0.57	3.6	50	0	32	20	2489	2671	2.4	0.3	34	29.2	45.2	5.7
	0.62	3.6	100	0	32	20	2489	0	2.4	0	34	0	45.7	5.2
[31]	0.65	3.3	0	0	19	20	0	2720	0	0.2	0	25	20.2	0
	0.65	3.2	25	0	19	20	2440	2720	5.8	0.2	33.6	25	18.5	0
	0.65	3.1	50	0	19	20	2440	2720	5.8	0.2	33.6	25	18	0
	0.65	3.1	75	0	19	20	2440	2720	5.8	0.2	0	25	16.5	0
	0.42	2.7	0	0	19	20	0	2720	0	0.2	33.6	25	40	0
	0.42	2.7	25	0	19	20	2440	2720	5.8	0.2	33.6	25	33	0
	0.42	2.6	50	0	19	20	2440	2720	5.8	0.2	33.6	25	34.5	0
	0.42	2.5	75	0	19	20	2440	2720	5.8	0.2	33.6	25	34	0
[32]	0.65	3.4	0	0	16	20	0	2730	0	2.5	0	0	31.9	0
	0.66	3.3	20	0	16	20	2400	2730	5	2.5	0	0	31.7	0
	0.68	3.1	50	0	16	20	2400	2730	5	2.5	0	0	32.4	0
	0.68	2.8	100	0	16	20	2400	0	5	0	0	0	30.1	0
	0.5	2.6	0	0	16	20	0	2730	0	2.5	0	0	44.8	0
	0.51	2.5	20	0	16	20	2400	2730	5	2.5	0	0	43.7	0
	0.53	2.3	50	0	16	20	2400	2730	5	2.5	0	0	37.5	0
	0.56	2.1	100	0	16	20	2400	0	5	0	0	0	40.5	0
[33]	0.45	1.9	0	0	19	25	2420	0	5.4	0.9	0	0	35.2	0
	0.45	3.4	64	0	19	25	2420	2740	5.4	0.9	0	0	41.4	0
	0.45	2.3	100	0	19	25	2420	2740	5.4	0	0	0	43.9	0

	0.45	2.1	0	0	19	25	2500	0	3.3	0.9	0	0	34.1	0
	0.45	3.1	64	0	19	25	2500	2740	3.3	0.9	0	0	44.8	0
	0.45	2.5	100	0	19	25	2500	2740	3.3	0	0	0	45.9	0
[34]	0.65	3.4	0	0	16	20	0	2720	0	2	0	23	31.9	0
	0.65	3.3	20	0	16	20	2400	2720	5	2	34	23	31.7	0
	0.65	3.1	50	0	16	20	2400	2720	5	2	34	23	32.4	0
	0.65	2.8	100	0	16	20	2400	0	5	0	34	0	30.1	0
	0.5	2.6	0	0	16	20	0	2720	0	2	0	23	44.8	0
	0.5	2.5	20	0	16	20	2400	2720	5	2	34	23	43.7	0
	0.5	2.8	50	0	16	20	2400	2720	5	2	34	23	37.5	0
	0.5	2.1	100	0	16	20	2400	0	5	0	34	0	40.5	0
[35]	0.43	3.1	0	0	20	20	0	2620	0	1.1	0	21.6	51.8	5.2
	0.43	3	25	0	20	20	2661	2620	1.9	1.1	0	21.6	47	4.2
	0.43	2.9	50	0	20	20	2602	2620	2.6	1.1	0	21.6	46	4.4
	0.43	2.8	100	0	20	20	2510	0	3.9	0	38.8	0	42.5	5
[36]	0.45	2.3	0	0	19	25	0	2730	0	0.45	0	23	44.4	0
	0.45	2.3	100	0	19	25	2490	0	4.8	0	37	0	41	0
	0.55	2.9	0	0	19	25	0	2730	0	0.45	0	23	36.7	0
	0.55	2.9	100	0	19	25	2490	0	4.8	0	37	0	33.3	0
	0.65	3.5	0	0	19	25	0	2730	0	0.45	0	23	30.4	0
	0.65	3.5	100	0	19	25	2490	0	4.8	0	37	0	24.8	0
[37]	0.6	4.6	0	0	19	20	0	0	0	0	0	0	25	0
	0.6	4.6	25	0	19	20	0	0	0	0	0	0	26.7	0
	0.6	4.5	50	0	19	20	0	0	0	0	0	0	21.5	0
	0.6	4.5	75	0	19	20	0	0	0	0	0	0	21.4	0
	0.6	4.4	100	0	19	20	0	0	0	0	0	0	20	0
	0.45	2.6	0	0	19	20	0	0	0	0	0	0	39.5	0
	0.45	2.6	25	0	19	20	0	0	0	0	0	0	38.3	0
	0.45	2.5	50	0	19	20	0	0	0	0	0	0	37	0
	0.45	2.5	75	0	19	19	0	0	0	0	0	0	35	0
	0.45	2.5	100	0	19	19	0	0	0	0	0	0	33.3	0
[38]	0.49	3.1	0	0	25	16	0	2710	0	0.8	0	0	44.3	0
	0.37	3	100	26.3	25	16	2490	0	2.9	0	0	0	37.6	0
	0.43	3	100	42.7	25	16	2570	0	2.9	0	0	0	43.3	0
	0.36	2.9	100	42.7	25	16	2440	0	5.6	0	0	0	42.6	0

	0.36	2.9	100	65.3	25	16	2470	0	5.3	0	0	0	44.7	0
[39]	0.53	6.5	0	0	32	15	0	2650	0	0.2	0	0	39.3	4.4
	0.43	5.4	100	0	32	15	2263	0	6	0	0	0	33.2	4.3
	0.49	5.1	100	0	32	15	2283	0	4.2	0	0	0	35.6	4.8
	0.53	5.1	100	0	32	15	2292	0	4.3	0	0	0	34.6	5
	0.6	5.3	100	0	32	15	2301	0	5	0	0	0	37.3	5.1
	0.54	6.4	90	0	32	15	2609	2650	1.5	0.2	0	0	45.4	4.4
	0.46	5.9	60	0	32	15	2518	2650	2.7	0.2	0	0	54.3	5.9
	0.44	5.8	60	0	32	15	2584	2650	1.6	0.2	0	0	54.4	6.4
	0.45	6.4	25	0	32	32	2594	2650	1.6	0.2	0	0	53.4	6
[40]	0.43	3	0	0	32	25	0	0	0	0	0	0	34.8	0
	0.47	2.9	30	0	32	32	0	0	0	0	0	0	31.9	0
	0.49	2.8	50	0	32	32	0	0	0	0	0	0	30.6	0
	0.54	2.7	100	0	32	32	0	0	0	0	0	0	29.7	0
[41]	0.66	4.6	0	0	20	22	0	2510	0	1.4	0	0	21	0
	0.66	4.6	30	0	20	22	2340	2510	5.3	1.4	0	0	20	0
	0.61	4.3	50	0	20	22	2340	2510	5.3	1.4	0	0	19	0
	0.58	4	100	0	20	22	2340	0	5.3	0	0	0	18	0
	0.55	3.8	0	0	20	22	0	2510	0	1.4	0	0	21	0
	0.55	3.8	30	0	20	22	2340	2510	5.3	1.4	0	0	23	0
	0.51	3.5	50	0	20	22	2340	2510	5.3	1.4	0	0	24	0
	0.48	3.4	100	0	20	22	2340	0	5.3	0	0	0	21	0
	0.5	3.5	0	0	20	22	0	2510	0	1.4	0	0	31	0
	0.5	3.5	30	0	20	22	2340	2510	5.3	1.4	0	0	25	0
	0.47	3.2	50	0	20	22	2340	2510	5.3	1.4	0	0	29	0
	0.44	3	100	0	20	22	2340	0	5.3	0	0	0	30	0
	0.48	3.3	0	0	20	22	0	2510	0	1.4	0	0	33	0
	0.48	3.3	30	0	20	12	2340	2510	5.3	1.4	0	0	39	0
	0.44	3.1	50	0	20	22	2340	2510	5.3	1.4	0	0	31	0
	0.42	2.9	100	0	20	22	2340	0	5.3	0	0	0	34	0
[42]	0.6	4.3	0	0	32	20	0	2381	0	0	0	0	36.6	0
	0.6	3.8	100	0	32	20	2264	0	2	0	0	0	33.6	0
	0.52	3.6	0	0	32	20	0	2389	0	0	0	0	41.8	0
	0.52	3.2	100	0	32	25	2276	0	2	0	0	0	41.1	0
	0.47	3	0	0	32	25	0	2387	0	0	0	0	48.6	0

	0.47	2.7	100	0	32	25	2273	0	2	0	0	0	48.1	0
[43]	0.6	3	0	37.3	12	20	0	2720	0	0.6	0	0	39.5	0
	0.59	3.2	10	37.3	12	20	2010	2720	10.9	0.6	0	0	40	0
	0.57	3.5	30	37.3	12	20	2010	2720	10.9	0.6	0	0	38.6	0
	0.54	3.8	50	37.3	12	20	2010	2720	10.9	0.6	0	0	37.6	0
	0.46	4.6	100	37.3	12	20	2010	0	10.9	0	0	0	38.6	0
	0.45	3.2	0	37.3	12	20	0	2720	0	0.6	0	0	53.3	0
	0.44	3.3	10	37.3	12	20	2010	2720	10.9	0.6	0	0	53.7	0
	0.42	3.7	30	37.3	12	20	2010	2720	10.9	0.6	0	0	51	0
	0.67	4	50	37.3	12	20	2010	2720	10.9	0.6	0	0	47.8	0
	0.68	4.8	100	37.3	12	20	2010	0	10.9	0	0	0	45.1	0
	0.67	3.3	0	37.3	12	20	0	2720	0	0.6	0	0	65.2	0
	0.7	3.4	10	37.3	12	20	2010	2720	10.9	0.6	0	0	64.6	0
	0.53	3.8	30	37.3	12	20	2010	2720	10.9	0.6	0	0	65.4	0
	0.53	4.1	50	37.3	12	20	2010	2720	10.9	0.6	0	0	63.2	0
	0.53	5	100	37.3	12	20	2010	0	10.9	0	0	0	63	0
[44]	0.54	3	0	41.4	20	22	0	2581	0	1.2	0	24.8	49.8	0
	0.54	3	20	41.4	20	22	2451	2581	7.3	1.2	40	24.8	50.5	0
	0.54	3	50	41.4	20	22	2451	2581	7.3	1.2	40	24.8	48.1	0
	0.54	2.9	100	41.4	20	22	2451	0	7.3	0	40	0	45.2	0
	0.45	3.1	0	41.4	20	22	0	2581	0	1.2	0	24.8	59.7	0
	0.45	3.1	20	41.4	20	22	2451	2581	7.3	1.2	40	24.8	64.7	0
	0.45	3.1	50	41.4	20	22	2451	2581	7.3	1.2	40	24.8	55	0
	0.45	3.1	100	41.4	20	22	2451	0	7.3	0	40	0	53.9	0
	0.4	3.2	0	41.4	20	22	0	2581	0	1.2	0	24.8	78.7	0
	0.4	3.2	20	41.4	20	22	2451	2581	7.3	1.2	40	24.8	69.9	0
	0.4	3.2	50	41.4	20	22	2451	2581	7.3	1.2	40	24.8	63.8	0
	0.4	3.1	100	41.4	20	22	2451	0	7.3	0	40	0	62.8	0
[45]	0.48	4.1	0	0	10	20	0	2670	0	1.5	0	11.9	38.9	5.8
	0.48	3.5	100	0	10	20	2360	0	4.7	0	15.1	0	38.6	5.2
	0.39	3.1	100	0	10	20	2280	0	6.2	0	22.1	0	38.1	5.2
	0.29	2.6	100	0	10	20	2220	0	7.8	0	25	0	39.3	5.2
	0.34	2.3	0	0	10	20	0	2670	0	1.5	0	11.9	61.9	5.2
	0.31	2.1	100	0	10	20	2360	0	4.7	0	15.1	0	60.1	5.8
	0.27	1.8	100	0	10	20	2280	0	6.2	0	22.1	0	60.2	5

	0.19	1.5	100	0	10	20	2220	0	7.8	0	25	0	62.8	4.4
[46]	0.52	3	100	0	25	20	2490	0	4.9	0	0	0	37.6	0
	0.52	3	100	0	25	20	2570	0	2.9	0	0	0	43.3	0
	0.52	2.9	100	0	25	20	2440	0	5.6	0	0	0	42.6	0
	0.52	2.9	100	0	25	32	2470	0	5.3	0	0	0	44.7	0
	0.52	3.1	0	0	25	32	0	2710	0	0.83	0	0	44.3	0
	0.58	3.2	0	0	32	16	0	2970	0	0.8	0	0	44.6	0
	0.52	3.2	53	0	32	16	2720	2970	4.8	0.8	0	0	41.4	0
[47]	0.58	3.2	100	0	32	16	2720	0	4.8	0	0	0	40.7	0
	0.52	3.2	54	0	32	25	2650	2970	4.6	0.8	0	0	38.3	0
	0.58	3.2	100	0	32	25	2650	0	4.6	0	0	0	36.6	0
	0.52	3.2	53	0	32	25	2880	2970	4.4	0.8	0	0	41.2	0
	0.58	3.2	100	0	32	25	2880	0	4.4	0	0	0	40.3	0
[48]	0.41	1.7	15	0	20	32	2330	2600	4.4	0.7	0	0	50.8	0
	0.41	1.7	30	0	20	32	2330	2600	4.4	0.7	0	0	44.9	0
	0.41	1.7	45	0	20	32	2330	2600	4.4	0.7	0	0	44.6	0
	0.41	1.7	60	0	20	32	2330	2600	4.4	0.7	0	0	42.4	0
	0.41	1.7	15	0	20	32	2370	2600	4	0.7	0	0	54	0
	0.41	1.7	30	0	20	32	2370	2600	4	0.7	0	0	56	0
	0.41	1.7	45	0	20	32	2370	2600	4	0.7	0	0	54.4	0
	0.41	1.7	60	0	20	32	2370	2600	4	0.7	0	0	40.6	0
	0.41	1.7	15	0	20	20	2390	2600	3.6	0.7	0	0	55.2	0
	0.41	1.7	30	0	20	20	2390	2600	3.6	0.7	0	0	53.5	0
	0.41	1.7	45	0	20	20	2390	2600	3.6	0.7	0	0	56.9	0
	0.41	1.7	60	0	20	20	2390	2600	3.6	0.7	0	0	54.7	0
	0.41	1.7	15	0	20	20	2320	2600	4.6	0.7	0	0	50.5	0
	0.41	1.7	30	0	20	20	2320	2600	4.6	0.7	0	0	48.9	0
	0.41	1.7	45	0	20	20	2320	2600	4.6	0.7	0	0	45.8	0
	0.41	1.7	60	0	20	20	2320	2600	4.6	0.7	0	0	40	0
	0.41	1.7	15	0	20	20	2390	2600	3.7	0.7	0	0	54.4	0
	0.41	1.7	30	0	20	20	2390	2600	3.7	0.7	0	0	50.2	0
	0.41	1.7	45	0	20	20	2390	2600	3.7	0.7	0	0	49.5	0
	0.41	1.7	60	0	20	20	2390	2600	3.7	0.7	0	0	40.4	0
	0.41	1.7	15	0	20	20	2390	2600	3.5	0.7	0	0	45	0
	0.41	1.7	30	0	20	20	2390	2600	3.5	0.7	0	0	46.9	0

	0.41	1.7	45	0	20	20	2390	2600	3.5	0.7	0	0	51.4	0
	0.41	1.7	60	0	20	20	2390	2600	3.5	0.7	0	0	53.2	0
	0.41	1.7	15	0	20	20	2380	2600	3.8	0.7	0	0	55.3	0
	0.41	1.7	30	0	20	20	2380	2600	3.8	0.7	0	0	55.9	0
	0.41	1.7	45	0	20	20	2380	2600	3.8	0.7	0	0	52.6	0
	0.41	1.7	60	0	20	20	2380	2600	3.8	0.7	0	0	48	0
	0.41	1.7	15	0	20	20	2380	2600	3.8	0.7	0	0	49.1	0
	0.41	1.7	30	0	20	20	2380	2600	3.8	0.7	0	0	49.9	0
	0.41	1.7	45	0	20	20	2380	2600	3.8	0.7	0	0	50.3	0
	0.41	1.7	60	0	20	20	2380	2600	3.8	0.7	0	0	47.5	0
	0.41	1.7	15	0	20	20	2400	2600	3.5	0.7	0	0	43.2	0
	0.41	1.7	30	0	20	20	2400	2600	3.5	0.7	0	0	53.7	0
	0.41	1.7	45	0	20	20	2400	2600	3.5	0.7	0	0	50	0
	0.41	1.7	60	0	20	20	2400	2600	3.5	0.7	0	0	43.3	0
	0.41	1.7	15	0	20	20	2370	2600	4	0.7	0	0	52.9	0
	0.41	1.7	30	0	20	20	2370	2600	4	0.7	0	0	49.9	0
	0.41	1.7	45	0	20	20	2370	2600	4	0.7	0	0	53.7	0
	0.41	1.7	60	0	20	20	2370	2600	4	0.7	0	0	46	0
[49]	0.48	5.1	0	36	25	20	0	2570	0	1.2	0	0	41.3	6.4
	0.48	5	27	36	25	20	2250	2570	7	1.2	0	0	51.4	5.8
	0.48	4.9	64	36	25	20	2250	2570	7	1.2	0	0	45.6	4.9
	0.48	5	37	36	25	20	2250	2570	7	1.2	0	0	44.7	4.8
	0.48	5	37	36	25	20	2250	2570	7	1.2	0	0	41.9	5.7
[50]	0.5	2.4	100	0	25	32	2452	0	4.1	0	0	0	51	0
	0.5	2.4	100	0	25	32	2452	0	4.1	0	0	0	49	0
	0.5	2.4	100	0	25	32	2452	0	4.1	0	0	0	48	0
	0.5	2.6	0	0	25	32	2452	2652	4.1	0.8	0	0	52	0
	0.5	2.5	50	0	25	32	2452	2652	4.1	0.8	0	0	51	0
	0.5	2.5	50	0	25	32	2452	2652	4.1	0.8	0	0	51	0
	0.5	2.5	50	0	25	32	2452	2652	4.1	0.8	0	0	51	0
	0.5	2.5	25	0	25	32	2452	2652	4.1	0.8	0	0	52	0
	0.5	2.5	25	0	25	25	2452	2652	4.1	0.8	0	0	50	0
	0.5	2.5	25	0	25	25	2452	2652	4.1	0.8	0	0	49	0
[51]	0.38	2	0	0	25	38	0	2630	0	1.2	0	0	54.1	7.7
	0.28	2	100	0	25	38	2260	0	7.5	0	0	0	38.3	6.5

	0.28	2	100	0	25	38	2260	0	7.5	0	0	0	32.9	5.9
	0.23	2	100	0	25	38	2260	0	7.5	0	0	0	33.2	5.5
	0.46	2.4	0	0	25	38	0	2630	0	1.2	0	0	42.2	7.1
	0.34	2.4	100	0	25	38	2260	0	7.5	0	0	0	31.3	5.7
	0.34	2.4	100	0	25	38	2260	0	7.5	0	0	0	28.4	5.3
	0.28	2.4	100	0	25	38	2260	0	7.5	0	0	0	28	5.1
	0.58	3.1	0	0	25	25	0	2630	0	1.2	0	0	28.8	5.9
	0.43	3.1	100	0	25	25	2260	0	7.5	0	0	0	26.5	5.5
	0.43	3.1	100	0	25	25	2260	0	7.5	0	0	0	23.3	5.1
	0.35	3.1	100	0	25	25	2260	0	7.5	0	0	0	21.6	4.7
	0.67	3.5	0	0	25	25	0	2630	0	1.2	0	0	23.6	5.2
	0.49	3.5	100	0	25	25	2260	0	7.5	0	0	0	21.6	5.3
	0.49	3.5	100	0	25	25	2260	0	7.5	0	0	0	18	4.8
	0.4	3.5	100	0	25	25	2260	0	7.5	0	0	0	18.8	4.5
	0.8	4.2	0	0	25	25	0	2630	0	1.2	0	0	17.3	4.4
	0.59	4.2	100	0	25	25	2260	0	7.5	0	0	0	16.1	5.2
	0.59	4.2	100	0	25	25	2260	0	7.5	0	0	0	13.4	4
	0.48	4.2	100	0	25	25	2260	0	7.5	0	0	0	13.9	3.9
[52]	0.6	3.6	0	0	20	20	0	2540	0	1.8	0	31	38	0
	0	0	0	0	0	0	0	0	0	0	0	0	0	0
	0.59	3.3	20	0	20	20	2320	2540	5.3	1.8	42	31	41	0
	0.57	3.3	50	0	20	20	2320	2540	5.3	1.8	42	31	44	0
	0.54	3	100	0	20	20	2320	0	5.3	0	42	31	45	0
	0.46	2.6	0	0	20	20	0	2540	0	1.8	42	0	51.5	0
	0.45	2.5	20	0	20	20	2320	2540	5.3	1.8	0	31	50.5	0
	0.44	2.5	50	0	20	20	2320	2540	5.3	1.8	42	31	45	0
	0.42	2.3	100	0	20	20	2320	0	5.3	0	42	0	56	0
	0.67	3.6	0	0	20	19	0	2540	0	1.8	0	31	37	0
	0.68	3.4	20	0	20	19	2320	2540	5.3	1.8	42	31	33.5	0
	0.67	3	50	0	20	19	2320	2540	5.3	1.8	42	31	32	0
	0.7	2.3	100	0	20	19	2320	0	5.3	0	42	0	32	0
	0.53	2.7	0	0	20	19	0	2540	0	1.8	0	31	45	0
	0.53	2.5	20	0	20	19	2320	2540	5.3	1.8	42	31	44	0
	0.53	2.2	50	0	20	19	2320	2540	5.3	1.8	42	31	41	0
	0.52	1.8	100	0	20	19	2320	0	5.3	0	42	0	41.5	0

	0.51	3.1	0	0	20	20	0	2540	0	1.8	0	31	46.5	0
	0.52	3.2	20	0	20	20	2320	2540	5.3	1.8	42	31	44	0
	0.54	3	50	0	20	20	2320	2540	5.3	1.8	42	31	41	0
	0.58	2.8	100	0	20	20	2320	0	5.3	0	42	0	33.5	0
	0.42	2.7	0	0	20	20	0	2540	0	1.8	0	31	58	0
	0.42	2.9	20	0	20	20	2320	2540	5.3	1.8	42	31	53.5	0
	0.44	2.7	50	0	20	20	2320	2540	5.3	1.8	42	31	54	0
	0.49	2.5	100	0	20	20	2320	0	5.3	0	42	0	40	0
[53]	0.42	2.6	50	0	20	19	2330	2590	6.1	1.2	34.6	29.1	41.6	0
	0.51	2.3	100	0	20	19	2330	0	6.1	0	34.6	0	31.4	0
	0.52	2.6	50	0	20	19	2330	2590	6.1	1.2	34.6	29.1	35.5	0
	0.61	2.3	100	0	20	19	2330	0	6.1	0	34.6	0	26	0
	0.44	2.6	50	0	20	19	2320	2590	5.8	1.2	32.2	29.1	44.6	0
	0.51	2.3	100	0	20	19	2320	0	5.8	0	32.2	0	36.7	0
	0.62	2.3	100	0	20	19	2320	0	5.8	0	32.2	0	29.5	0
	0.41	2.8	20	0	20	19	2360	2590	3.9	1.2	30.8	29.1	46.1	0
	0.42	2.6	50	0	20	20	2360	2590	3.9	1.2	30.8	29.1	45.1	0
	0.45	2.3	100	0	20	20	2360	0	3.9	0	30.8	0	42.9	0
	0.5	2.8	20	0	20	20	2360	2590	3.9	1.2	30.8	29.1	39.3	0
	0.52	2.6	50	0	20	20	2360	2590	3.9	1.2	30.8	29.1	39.5	0
	0.54	2.3	100	0	20	20	2360	0	3.9	0	30.8	0	37.7	0
	0.42	2.8	20	0	20	20	2350	2590	4.5	1.2	28.5	29.1	48.1	0
	0.43	2.6	50	0	20	20	2350	2590	4.5	1.2	28.5	29.1	41	0
	0.4	2.3	100	0	20	20	2350	0	4.5	0	28.5	0	38.7	0
	0.51	2.8	20	0	20	20	2350	2590	4.5	1.2	28.5	29.1	42.7	0
	0.52	2.6	50	0	20	20	2350	2590	4.5	1.2	28.5	29.1	35.4	0
	0.5	2.3	100	0	20	20	2350	0	4.5	0	28.5	0	31.4	0
	0.42	2.8	20	0	20	20	2350	2590	4.7	1.2	30.1	29.1	48.5	0
	0.42	2.6	50	0	20	20	2350	2590	4.7	1.2	30.1	29.1	45.4	0
	0.43	2.3	100	0	20	20	2350	0	4.7	0	30.1	0	37	0
	0.52	2.8	20	0	20	20	2350	2590	4.7	1.2	30.1	29.1	41.3	0
	0.52	2.6	50	0	20	20	2350	2590	4.7	1.2	30.1	29.1	36.8	0
	0.56	2.3	100	0	20	20	2350	0	4.7	0	30.1	0	31.2	0
[54]	0.41	2.6	0	0	32	20	0	2820	0	0.4	0	0	47.2	0
	0.38	2.6	33	0	32	20	2578	2820	9.3	0.4	0	0	42.4	0

	0.36	2.6	53	0	32	20	2578	2820	9.3	0.4	0	0	45.7	0
	0.34	2.6	72	0	32	20	2578	2820	9.3	0.4	0	0	36.7	0
	0.31	2.6	100	0	32	20	2578	0	9.3	0	0	0	38.9	0
[55]	0.47	3.8	0	0	20	25	0	2610	0	1	0	0	53.1	0
	0.47	3.7	20	0	20	25	2336	2610	3.6	1	0	0	50	0
	0.47	3.6	50	0	20	25	2315	2610	3.6	1	0	0	45.3	0
	0.47	3.6	75	0	20	25	2295	2610	3.6	1	0	0	44	0
	0.47	3.5	100	0	20	25	2273	0	3.6	0	0	0	41.6	0
[56]	0.29	2.9	0	0	10	10	0	2680	0	2.1	0	24.8	102.1	6.5
	0.29	2.8	20	100	10	10	2470	2680	3.7	2.1	24	24.8	108	7.4
	0.29	2.8	50	100	10	10	2470	2680	3.7	2.1	24	24.8	104.8	7.7
	0.29	2.7	100	100	10	10	2470	0	3.7	0	24	0	108.5	6.8
	0.29	2.8	20	60	10	10	2390	2680	4.9	2.1	25.2	24.8	102.5	8
	0.29	2.7	50	60	10	10	2390	2680	4.9	2.1	25.2	24.8	103.1	6.8
	0.29	2.6	100	60	10	10	2390	0	4.9	0	25.2	0	100.8	6.3
	0.29	2.8	20	40	10	10	2300	2680	5.9	2.1	24.3	24.8	104.3	6.7
	0.29	2.7	50	40	10	10	2300	2680	5.9	2.1	24.3	24.8	96.8	6.8
	0.29	2.5	100	40	10	10	2300	0	5.9	0	24.3	0	91.2	6.5
[57]	0.65	4.6	0	0	20	30	0	2680	0	1.53	0	20	18	2.4
	0.65	4.7	25	0	20	30	2380	2680	6.94	1.53	29	20	14.7	2.1
	0.65	4.8	50	0	20	30	2380	2680	6.94	1.53	29	20	14.6	1.9
	0.65	4.8	75	0	20	30	2380	2680	6.94	1.53	29	20	14.2	1.9
	0.72	5.8	0	0	20	20	0	2680	0	1.53	0	20	30.8	2.3
	0.72	5.9	20	0	20	20	2380	2680	6.94	1.53	29	20	26.8	2.7
	0.72	6	40	0	20	20	2380	2680	6.94	1.53	29	20	26.6	2
	0.45	1.9	0	0	16	20	0	2680	0	1.53	0	20	66.9	3.8
	0.45	2.3	20	0	16	20	2380	2680	6.94	1.53	29	20	49.3	2.8
	0.45	2.5	40	0	16	20	2380	2680	6.94	1.53	29	20	40.9	2.7
[58]	0.6	3.5	0	0	16	20	0	2680	0	1.9	0	0	42	4.7
	0.6	3.4	20	0	16	20	2380	2680	6.9	1.9	0	0	42.9	4.7
	0.6	3.4	50	0	16	20	2380	2680	6.9	1.9	0	0	42.5	4.7
	0.6	3.2	100	0	16	20	2380	0	6.9	0	0	0	40.9	4.8
	0.5	2.7	0	0	16	20	0	2680	0	1.9	0	0	50.2	5.1
	0.5	2.6	20	0	16	20	2380	2680	6.9	1.9	0	0	51.6	5.1
	0.5	2.5	50	0	16	20	2380	2680	6.9	1.9	0	0	51.6	5.1

	0.5	2.4	100	0	16	20	2380	0	6.9	0	0	0	50.3	5.3
[59]	0.5	3.4	0	0	22	22	0	2670	0	0	0	0	46.7	0
	0.5	3.4	50	0	12	22	2380	2670	0	0	0	0	46.9	0
	0.5	3.4	50	0	22	22	2380	2670	0	0	0	0	46.4	0
	0.5	3.4	100	0	22	22	2380	0	0	0	0	0	48.6	0
[60]	0.52	2.2	0	0	19	10	0	2810	0	0.4	0	0	29.9	3.6
	0.49	2.1	25	0	19	10	2500	2810	6.6	0.4	0	0	32.6	3.3
[61]	0.5	3.5	50	0	8	20	2330	2750	3.8	0.8	41.4	24.3	33	0
	0.5	3.2	50	0	8	20	2280	0	4.1	0	0	0	29.1	0
[62]	0.68	3.8	0	0	20	20	0	2600	0	0.9	0	0	34.5	0
	0.68	3.6	100	0	20	20	2450	0	3.1	0	0	0	35	0
	0.68	3.4	100	0	20	20	2370	0	7.1	0	0	0	29.2	0
	0.68	3.4	100	0	20	20	2360	0	7.8	0	0	0	27.7	0
	0.51	3.3	0	0	20	20	0	2600	0	0.9	0	0	48.3	0
	0.51	3.1	100	0	20	20	2450	0	3.1	0	0	0	47.6	0
	0.51	3	100	0	20	20	2370	0	7.1	0	0	0	42	0
	0.51	3	100	0	20	20	2360	0	7.8	0	0	0	42.9	0
	0.44	2.5	0	0	20	20	0	2600	0	0.9	0	0	61.6	0
	0.44	2.4	100	0	20	20	2450	0	3.1	0	0	0	60	0
	0.44	2.3	100	0	20	20	2370	0	7.1	0	0	0	53.7	0
	0.44	2.3	100	0	20	20	2360	0	7.8	0	0	0	53.2	0
	0.34	2.2	0	0	20	20	0	2600	0	0.9	0	0	80.8	0
	0.34	2.1	100	0	20	20	2450	0	3.1	0	0	0	78.2	0
	0.34	2	100	0	20	20	2370	0	7.1	0	0	0	71.2	0
	0.34	2	100	0	20	20	2360	0	7.8	0	0	0	65.4	0
[63]	0.5	3.1	0	0	19	19	0	2730	0	0.3	0	0	36.5	0
	0.5	3	30	0	19	19	2570	2730	2.7	0.3	0	0	33.6	0
	0.5	3	60	0	19	19	2570	2730	2.7	0.3	0	0	30.4	0
	0.5	2.8	100	0	19	19	2570	0	2.7	0	0	0	29.1	0
[64]	0.65	3.1	0	0	20	19	0	2500	0	1.7	0	32	40.5	0
	0.65	3.2	20	0	20	19	2300	2500	5.2	1.7	40.2	32	39.5	0
	0.65	3.2	50	0	20	19	2300	2500	5.2	1.7	40.2	0	40.8	0
	0.65	3.2	100	0	20	19	2300	0	5.2	0	40.2	32	43.7	0
	0.65	3.1	0	0	20	19	0	2500	0	1.7	0	32	40.5	0
	0.65	3.1	20	0	20	19	2300	2500	5.5	1.7	28.6	32	41	0

	0.65	3.1	50	0	20	19	2300	2500	5.5	1.7	28.6	32	38.8	0
	0.65	3.2	100	0	20	19	2300	0	5.5	0	28.6	0	39.9	0
[65]	0.42	2.7	0	0	25	25	0	2570	0	1.1	0	0	38.6	10.2
	0.4	2.7	16	0	25	25	2200	2570	5.4	1.1	0	0	32.7	9.7
	0.39	2.2	37	0	25	25	2200	2570	5.4	1.1	0	0	31.7	9
	0.36	2.7	52	0	25	20	2200	2570	5.4	1.1	0	0	29	8.9
[66]	0.86	4.6	0	0	22	20	0	2537	0	1.3	0	0	23.9	0
	0.65	3.4	0	0	22	20	0	2537	0	1.3	0	0	38.7	0
	0.41	2.9	0	0	22	20	0	2537	0	1.3	0	0	71.1	0
	0.87	4.6	100	0	22	20	2451	0	7.8	0	0	0	19.7	0
	0.66	3.4	100	0	22	20	2387	0	6.9	0	0	0	35.7	0
	0.42	2.8	100	0	22	20	2362	0	4.2	0	0	0	66.8	0
	0.86	4.6	100	0	22	20	2456	0	7.5	0	0	0	21.8	0
	0.65	3.5	100	0	22	20	2455	0	6.4	0	0	0	36.1	0
	0.42	2.9	100	0	22	20	2496	0	4.2	0	0	0	68.5	0
	0.81	4.9	0	0	22	20	0	2665	0	1	0	0	27.5	0
	0.63	3.6	0	0	22	20	0	2665	0	1	0	0	42.4	0
	0.4	3	0	0	22	20	0	2665	0	1	0	0	72.3	0
	0.84	4.5	100	0	22	20	2401	0	7.6	0	0	0	21	0
	0.63	3.5	100	0	22	20	2484	0	5.4	0	0	0	41.1	0
	0.4	2.8	100	0	22	20	2363	0	3.6	0	0	0	70.2	0
	0.82	4.7	100	0	22	20	2447	0	6.9	0	0	0	23.6	0
	0.64	3.4	100	0	22	22	2458	0	5.8	0	0	0	39.7	0
	0.42	2.9	100	0	22	22	2464	0	3.9	0	0	0	66.5	0
[67]	0.64	3	0	0	19	20	0	2634	0	1.3	0	0	33	0
	0.77	3.1	100	0	19	20	2268	0	4.9	0	0	0	27.5	0
	0.7	3.4	100	0	19	20	1946	0	11.9	0	0	0	29.9	0
[68]	0.6	3.6	0	0	19	19	0	2730	0	0.5	0	23	47.8	0
	0.59	3.3	20	0	19	19	2320	2730	5.3	0.5	37	23	49.3	0
	0.57	3.3	50	0	19	19	2320	2730	5.3	0.5	37	23	47.5	0
	0.54	3	100	0	19	19	2320	0	5.3	0	37	0	53.7	0
	0.46	2.6	0	0	19	19	0	2730	0	0.5	0	23	62	0
	0.45	2.5	20	0	19	19	2320	2730	5.3	0.5	37	23	64.8	0
	0.44	2.5	50	0	19	19	2320	2730	5.3	0.5	37	23	63.5	0
	0.42	2.3	100	0	19	19	2320	0	5.3	0	37	0	65.1	0

0.67	3.6	0	0	19	19	0	2730	0	0.5	0	23	62	0
0.68	3.4	20	0	19	19	2320	2730	5.3	0.5	37	23	64.8	0
0.67	3	50	0	19	19	2320	2730	5.3	0.5	37	23	63.5	0
0.7	2.3	100	0	19	19	2320	0	5.3	0	37	0	65.1	0
0.53	2.7	0	0	19	19	0	2730	0	0.5	0	23	57.3	0
0.53	2.5	20	0	19	19	2320	2730	5.3	0.5	37	23	54.9	0
0.53	2.2	50	0	19	19	2320	2730	5.3	0.5	37	23	51.5	0
0.52	1.8	100	0	19	19	2320	0	5.3	0	37	0	50.3	0
0.51	3.1	0	0	19	19	0	2730	0	0.5	0	23	60.1	0
0.52	3.2	20	0	19	19	2320	2730	5.3	0.5	37	23	56.5	0
0.54	3	50	0	19	19	2320	2730	5.3	0.5	37	23	48.9	0
0.58	2.8	100	0	19	19	2320	0	5.3	0	37	0	43.1	0
0.42	2.7	0	0	19	19	0	2730	0	0.5	0	23	72.9	0
0.42	2.9	20	0	19	19	2320	2730	5.3	0.5	37	23	67.4	0
0.44	2.7	50	0	19	19	2320	2730	5.3	0.5	37	23	61.2	0
0.49	2.5	100	0	19	19	2320	0	5.3	0	37	0	53.7	0

References

1. Yoda, K., Yoshikane, T., Nakashima, Y., Soshiroda, T. *Recycled cement and recycled concrete in Japan*. In: Proceedings of the international conference on demolition and reuse of concrete and masonry; 7–11 November 1988, Tokyo, Japan, 527–536.
2. Limbachiya, M.C.; Leelawat, T.; Dhir, R.K. Use of recycled concrete aggregate in high-strength concrete. *Mater. Struct.* **2000**, *33*, 574–580, doi:10.1007/bf02480538.
3. Ajdukiewicz, A.; Kliszczewicz, A. Influence of recycled aggregates on mechanical properties of HS/HPC. *Cem. Concr. Compos.* **2002**, *24*, 269–279, doi:10.1016/s0958-9465(01)00012-9.
4. Gómez-Soberón, J.M. Porosity of recycled concrete with substitution of recycled concrete aggregate: An experimental study. *Cem. Concr. Res.* **2002**, *32*, 1301–1311, doi:10.1016/s0008-8846(02)00795-0.
5. Vázquez, E.; Hendriks, C.F.; Janssen, G.M.T. *Influence of recycled concrete aggregates on concrete durability*. In *International RILEM Conference on the Use of Recycled Materials in Building and Structures;* RILEM Publications SARL: Paris, France, 2004, 554–562.
6. Poon, C.; Shui, Z.; Lam, L.; Fok, H.; Kou, S. Influence of moisture states of natural and recycled aggregates on the slump and compressive strength of concrete. *Cem. Concr. Res.* **2004**, *34*, 31–36, doi:10.1016/s0008-8846(03)00186-8.
7. Lin, Y.-H.; Tyan, Y.-Y.; Chang, T.-P.; Chang, C.-Y. An assessment of optimal mixture for concrete made with recycled concrete aggregates. *Cem. Concr. Res.* **2004**, *34*, 1373–1380, doi:10.1016/j.cemconres.2003.12.032.
8. Xiao, J.-Z.; Li, J.; Zhang, C. On relationships between the mechanical properties of recycled aggregate concrete: An overview. *Mater. Struct.* **2006**, *39*, 655–664, doi:10.1617/s11527-006-9093-0.
9. X.U. Wei, Experimental study on influence of recycled coarse aggregates contents on properties of recycled aggregate concrete, Concrete 10 (2006) 45-47.
10. Etxeberria, M.; Marí, A.R.; Vázquez, E. Recycled aggregate concrete as structural material. *Mater. Struct.* **2007** *40*, 529-541.
11. Etxeberria, M.; Vázquez, E.; Mari, A.; Barra, M. Influence of amount of recycled coarse aggregates and production process on properties of recycled aggregate concrete. *Cem. Concr. Res.* **2007**, *37*, 735–742, doi:10.1016/j.cemconres.2007.02.002.
12. Evangelista, L.; de Brito, J. Mechanical behaviour of concrete made with fine recycled concrete aggregates. *Cem. Concr. Compos.* **2007**, *29*, 397–401, doi:10.1016/j.cemconcomp.2006.12.004.
13. Poon, C.S.; Kou, S.C.; Lam, L. Influence of recycled aggregate on slump and bleeding of fresh concrete. *Mater. Struct.* **2006**, *40*, 981–988, doi:10.1617/s11527-006-9192-y.
14. Ajdukiewicz, A.B.; Kliszczewicz, A.T.; Comparative tests of beams and columns made of recycled aggregate concrete and natural aggregate concrete. *J. Adv. Concr. Technol.* **2007**, *5*, 259–273.
15. Min-Ping, H.U. Mechanical properties of concrete prepared with different recycled coarse aggregates replacement rate, *Concrete* **2007,** *2*, 16.
16. Kou, S.C.; Poon, C.S.; Chan, D. Influence of Fly Ash as Cement Replacement on the Properties of Recycled Aggregate Concrete. *J. Mater. Civ. Eng.* **2007**, *19*, 709–717, doi:10.1061/(asce)0899-1561(2007)19:9(709).
17. Rahal, K. Mechanical properties of concrete with recycled coarse aggregate. *Build. Environ.* **2007**, *42*, 407–415, doi:10.1016/j.buildenv.2005.07.033.
18. Wang, Z.W. Production and properties of high quality recycled aggregates. *Concrete* **2007,** *3*, 74–77.
19. Casuccio, M.; Torrijos, M.; Giaccio, G.; Zerbino, R. Failure mechanism of recycled aggregate concrete. *Constr. Build. Mater.* **2008**, *22*, 1500–1506, doi:10.1016/j.conbuildmat.2007.03.032.
20. Min-Ping, H. Mechanical properties of recycled aggregate concrete at early ages. *Concrete* **2008**, *223*, 37–41.
21. Kou, S.C.; Poon, C.S.; Chan, D. Influence of fly ash as a cement addition on the hardened properties of recycled aggregate concrete. *Mater. Struct.* **2007**, *41*, 1191–1201, doi:10.1617/s11527-007-9317.
22. Yang, K.-H.; Chung, H.-S.; Ashour, A.F. Influence of Type and Replacement Level of Recycled Aggregates on Concrete Properties, *ACI Mater. J.* **2008**, *105*, 289–296.
23. Zhou, H.; Liu, B.K.; Lu, G. Experimental research on the basic mechanical properties of recycled aggregate concrete. *J Anhui Inst Architect Indust* **2008**, *16*, 4–8.
24. Domingo-Cabo, A.; Lázaro, C.; Gayarre, F.L.; Serrano-López, M.; Serna, P.; Castaño-Tabares, J. Creep and shrinkage of recycled aggregate concrete. *Constr. Build. Mater.* **2009**, *23*, 2545–2553, doi:10.1016/j.conbuildmat.2009.02.018.
25. Padmini, A.K.; Ramamurthy, K.; Mathews, M.S.; Influence of parent concrete on the properties of recycled aggregate concrete. *Constr. Build. Mater.* **2009**, *23*, 829–836.
26. Yang, X.; Wu, J.; Liang, J.G. Experimental study on relationship between tensile strength and compressive strength of recycled aggregate concrete. *Sichuan Build. Sci* **2009**, *35*, 190–192.
27. Ye, H. Experimental study on mechanical properties of concrete made with high quality recycled aggregates. *Sichuan Build Sci* **2009**, *35*, 195–199.
28. Corinaldesi, V. Mechanical and elastic behaviour of concretes made of recycled-concrete coarse aggregates. *Constr. Build. Mater.* **2010**, *24*, 1616–1620, doi:10.1016/j.conbuildmat.2010.02.031.
29. Kumutha, R.; Vijai, K. Strength of concrete incorporating aggregates recycled from demolition waste. *ARPN J. of Eng. Appl. Sci.* **2010**, *5*, 64–71.

30. Malešev, M.; Radonjanin, V.; Marinković, S. Recycled Concrete as Aggregate for Structural Concrete Production. *Sustainability* **2010**, *2*, 1204–1225, doi:10.3390/su2051204.
31. Zega, C.J.; Di Maio, Ángel A. Recycled Concretes Made with Waste Ready-Mix Concrete as Coarse Aggregate. *J. Mater. Civ. Eng.* **2011**, *23*, 281–286, doi:10.1061/(asce)mt.1943-5533.0000165.
32. Belén, G.-F.; Fernando, M.-A.; Diego, C.L.; Sindy, S.-P. Stress–strain relationship in axial compression for concrete using recycled saturated coarse aggregate. *Constr. Build. Mater.* **2011**, *25*, 2335–2342, doi:10.1016/j.conbuildmat.2010.11.031.
33. Fathifazl, G.; Razaqpur, A.G.; Isgor, O.B.; Abbas, A.; Fournier, B.; Foo, S.; Creep and drying shrinkage characteristics of concrete produced with coarse recycled concrete aggregate. *Cem. Concr. Compos.* **2011**, *33*, 1026–1037.
34. González-Fonteboa, B.; Martínez-Abella, F.; Eiras-López, J.; Paz, S.S. Effect of recycled coarse aggregate on damage of recycled concrete. *Mater. Struct.* **2011**, *44*, 1759–1771, doi:10.1617/s11527-011-9736-7.
35. Rao, M.C.; Bhattacharyya, S.K.; Barai, S.V. Influence of field recycled coarse aggregate on properties of concrete. *Mater. Struct.* **2010**, *44*, 205–220, doi:10.1617/s11527-010-9620-x.
36. Somna, R.; Jaturapitakkul, C.; Chalee, W.; Rattanachu, P. Effect of the Water to Binder Ratio and Ground Fly Ash on Properties of Recycled Aggregate Concrete. *J. Mater. Civ. Eng.* **2012**, *24*, 16–22, doi:10.1061/(asce)mt.1943-5533.0000360.
37. Elhakam, A.A.; Mohamed, A.E.; Awad, E. Influence of self-healing, mixing method and adding silica fume on mechanical properties of recycled aggregates concrete. *Constr. Build. Mater.* **2012**, *35*, 421–427, doi:10.1016/j.conbuildmat.2012.04.013.
38. Cui, Z.L.; Lu, S.S.; Wang, Z.S. Influence of recycled aggregate on strength and anti-carbonation properties of recycled aggregate concrete. *J. Build. Mater* **2012,** *15*, 264–267.
39. Hoffmann, C.; Schubert, S.; Leemann, A.; Motavalli, M. Recycled concrete and mixed rubble as aggregates: Influence of variations in composition on the concrete properties and their use as structural material. *Constr. Build. Mater.* **2012**, *35*, 701–709.
40. Li, H.; Xiao, J.Z. On fatigue strength of recycled aggregate concrete based on its elastic modulus. *J. Build. Mater* **2012**, *15*, 260–263.
41. Limbachiya, M.; Meddah, M.S.; Ouchagour, Y. Performance of Portland/Silica Fume Cement Concrete Produced with Recycled Concrete Aggregate. ACI Mater. J. **2012**, *109*, 91–100.
42. Marinković, S.; Radonjanin, V.; Malešev, M.; Ignjatović, I. Comparative environmental assessment of natural and recycled aggregate concrete. *Waste Manag.* **2010**, *30*, 2255–2264, doi:10.1016/j.wasman.2010.04.012.
43. Pereira, P.; Evangelista, L.; de Brito, J. The effect of superplasticizers on the mechanical performance of concrete made with fine recycled concrete aggregates. *Cem. Concr. Compos.* **2012**, *34*, 1044–1052, doi:10.1016/j.cemconcomp.2012.06.009.
44. Barbudo, A.; De Brito, J.; Evangelista, L.; Bravo, M.; Agrela, F. Influence of water-reducing admixtures on the mechanical performance of recycled concrete. *J. of Clean. Prod.* **2013**, *59*, 93–98.
45. Butler, L.; West, J.S.; Tighe, S.L.; Effect of recycled concrete coarse aggregate from multiple sources on the hardened properties of concrete with equivalent compressive strength. *Constr. Build. Mater.* **2013**, *47*, 1292–1301.
46. Chen, Z.P.; Xu, J.J.; Zheng, H.H.; Su, Y.S.; Xue, J.Y.; Li, J.T. Basic mechanical properties test and stress-strain constitutive relations of recycled coarse aggregate concrete. *J. Build. Mater.* **2013**, *16*, 24–32.
47. Y.L. Hou, G. Zheng, Mechanical properties of recycled aggregate concrete in different age. *J Build Mater* **2013**, *16*, 683–687.
48. Ismail, S.; Ramli, M. Engineering properties of treated recycled concrete aggregate (RCA) for structural applications. *Constr. Build. Mater.* **2013**, *44*, 464–476.
49. Manzi, S.; Mazzotti, C.; Bignozzi, M.C. Short and long-term behavior of structural concrete with recycled concrete aggregate. *Cem. Concr. Compos.* **2013**, *37*, 312–318.
50. Matias, D.; De Brito, J.; Rosa, A.; Pedro, D. Mechanical properties of concrete produced with recycled coarse aggregates–Influence of the use of superplasticizers. *Constr. Build. Mater.* **2013**, *44,* 101–109.
51. Sheen, Y.-N.; Wang, H.-Y.; Juang, Y.-P.; Le, D.-H. Assessment on the engineering properties of ready-mixed concrete using recycled aggregates. *Constr. Build. Mater.* **2013**, *45*, 298–305.
52. Thomas, C.; Setién, J.; Polanco, J.A.; Alaejos, P.; De Juan, M.S. Durability of recycled aggregate concrete. *Constr. Build. Mater.* **2013**, *40*, 1054–1065, doi:10.1016/j.conbuildmat.2012.11.106.
53. Ulloa, V.A.; García-Taengua, E.; Pelufo, M.-J.; Domingo, A.; Serna, P. New views on effect of recycled aggregates on concrete compressive strength. ACI Mater. J. **2013**, *110*, 1–10.
54. Xiao, J.; Li, H.; Yang, Z. Fatigue behavior of recycled aggregate concrete under compression and bending cyclic loadings. *Constr. Build. Mater.* **2013**, *38*, 681–688, doi:10.1016/j.conbuildmat.2012.09.024.
55. Younis, K.; Pilakoutas, K. Strength prediction model and methods for improving recycled aggregate concrete. *Constr. Build. Mater.* **2013**, *49*, 688–701, doi:10.1016/j.conbuildmat.2013.09.003.
56. Andreu, G.; Miren, E. Experimental analysis of properties of high performance recycled aggregate concrete. *Constr. Build. Mater.* **2014**, *52*, 227–235, doi:10.1016/j.conbuildmat.2013.11.054.

57. Beltrán, M.G.; Agrela, F.; Barbudo, A.; Ayuso, J.; Ramírez, A. Mechanical and durability properties of concretes manufactured with biomass bottom ash and recycled coarse aggregates. *Constr. Build. Mater.* **2014**, *72*, 231–238, doi:10.1016/j.conbuildmat.2014.09.019.
58. Beltrán, M.G.; Barbudo, A.; Agrela, F.; Galvín, A.P.; Jiménez, J.R. Effect of cement addition on the properties of recycled concretes to reach control concretes strengths. *J. Clean. Prod.* **2014**, *79*, 124–133, doi:10.1016/j.jclepro.2014.05.053.
59. Çakır, Ö.; Sofyanlı, Ö.Ö. Influence of silica fume on mechanical and physical properties of recycled aggregate concrete. *HBRC J.* **2015**, *11*, 157–166, doi:10.1016/j.hbrcj.2014.06.002.
60. Carneiro, J.A.; Lima, P.R.L.; Leite, M.B.; Toledo Filho, R.D. Compressive stress–strain behavior of steel fiber reinforced-recycled aggregate concrete. *Cem. Concr. Compos.* **2014**, *46*, 65–72.
61. Dilbas, H.; Şimşek, M.; Çakır Ö. An investigation on mechanical and physical properties of recycled aggregate concrete (RAC) with and without silica fume. *Constr. Build. Mater.* **2014**, *61*, 50–59, doi:10.1016/j.conbuildmat.2014.02.057.
62. Duan, Z.; Poon, C.S. Properties of recycled aggregate concrete made with recycled aggregates with different amounts of old adhered mortars. *Mater. Des.* **2014**, *58*, 19–29, doi:10.1016/j.matdes.2014.01.044.
63. Folino, P.; Xargay, H. Recycled aggregate concrete – Mechanical behavior under uniaxial and triaxial compression. *Constr. Build. Mater.* **2014**, *56*, 21–31, doi:10.1016/j.conbuildmat.2014.01.073.
64. Gayarre, F.L.; Pérez, C.L.-C.; López, M.A.S.; Cabo, A.D. The effect of curing conditions on the compressive strength of recycled aggregate concrete. *Constr. Build. Mater.* **2014**, *53*, 260–266, doi:10.1016/j.conbuildmat.2013.11.112.
65. Kang, T.H.-K.; Kim, W.; KwakY.-K.; Hong, S.-G. Flexural Testing of Reinforced Concrete Beams with Recycled Concrete Aggregates. *ACI Struct. J.* **2014**, *111*, doi:10.14359/51686622.
66. Pedro, D.; de Brito, J.; Evangelista, L. Performance of concrete made with aggregates recycled from precasting industry waste: influence of the crushing process. *Mater. Struct.* **2014**, *48*, 3965–3978, doi:10.1617/s11527-014-0456-7.
67. Pepe, M.; Toledo Filho, R.D.; Koenders, E.A.B.; Martinelli, E. Alternative processing procedures for recycled aggregates in structural concrete. *Constr. and Build. Mater.* **2014,** *69,* 124–132.
68. Thomas, C.; Sosa, I.; Setién, J.; Polanco, J.A.; Cimentada, A.I. Evaluation of the fatigue behavior of recycled aggregate concrete. *J. Clean. Prod.* **2014**, *65*, 397–405.
